# Shared Signature Dynamics Tempered by Local Fluctuations Enables Fold Adaptability and Specificity

**DOI:** 10.1093/molbev/msz102

**Published:** 2019-04-27

**Authors:** She Zhang, Hongchun Li, James M Krieger, Ivet Bahar

**Affiliations:** Department of Computational and Systems Biology, School of Medicine, University of Pittsburgh, Pittsburgh, PA

**Keywords:** protein dynamics, evolution, superfamily, elastic network model, LeuT, PBP, TIM barrel, CATH, *ProDy*

## Abstract

Recent studies have drawn attention to the evolution of protein dynamics, in addition to sequence and structure, based on the premise structure-encodes-dynamics-encodes-function. Of interest is to understand how functional differentiation is accomplished while maintaining the fold, or how intrinsic dynamics plays out in the evolution of structural variations and functional specificity. We performed a systematic computational analysis of 26,899 proteins belonging to 116 CATH superfamilies. Characterizing cooperative mechanisms and convergent/divergent features that underlie the shared/differentiated dynamics of family members required a methodology that lends itself to efficient analyses of large ensembles of proteins. We therefore introduced, *SignDy*, an integrated pipeline for evaluating the *signature dynamics* of families based on elastic network models. Our analysis confirmed that family members share conserved, highly cooperative (*global*) modes of motion. Importantly, our analysis discloses a subset of motions that sharply distinguishes *subfamilies*, which lie in a low-to-intermediate frequency regime of the mode spectrum. This regime has maximal impact on functional differentiation of families into subfamilies, while being evolutionarily conserved among subfamily members. Notably, the high-frequency end of the spectrum also reveals evolutionary conserved features across and within subfamilies; but in sharp contrast to global motions, high-frequency modes are minimally collective. Modulation of robust/conserved global dynamics by low-to-intermediate frequency fluctuations thus emerges as a versatile mechanism ensuring the adaptability of selected folds and the specificity of their subfamilies. *SignDy* further allows for dynamics-based categorization as a new layer of information relevant to distinctive mechanisms of action of subfamilies, beyond sequence or structural classifications.

## Introduction

Studies in recent years have established the role of structural dynamics, also called *intrinsic dynamics*, in facilitating, if not driving, the interactions and function of biomolecular systems in the cell. Many biological events, including substrate recognition, binding and transport, allosteric signaling, communication and regulation, and mechanochemical responses, shortly referred to as *protein actions*, take advantage of the proteins’ *intrinsic dynamics* (Bahar et al. 2017). Intrinsic dynamics refers to collective thermal fluctuations in the conformational space, uniquely defined by the 3D architecture, or fold, under physiological conditions. Among the spectrum of motions intrinsically accessible to a structure, the modes of motions with the lowest frequency, called *global modes*, are often distinguished by their cooperativity and easy accessibility, hence their involvement in allosteric responses (Townsend et al. 2015), and qualification as *soft modes*.

Rapid evaluation of intrinsic dynamics with the help of elastic network models (ENMs) introduced around the turn of the century (Tirion 1996; Bahar et al. 1997; Hinsen et al. 2000; Atilgan et al. 2001) has enabled a deeper understanding of the functional significance of global motions (Tama and Sanejouand 2001; Ma 2005; Delarue 2008; Zheng et al. 2009; Fuglebakk et al. 2012, 2015; Tirion 2015; Hsieh et al. 2016; Lopez-Blanco and Chacon 2016). ENMs present the important advantage of yielding a unique analytical solution for the collective dynamics of each structure, thus overcoming the sampling inaccuracies or computational time/memory limitations of conventional molecular dynamics simulations (Dror et al. 2012; Luitz et al. 2015; Bottaro and Lindorff-Larsen 2018; Srivastava et al. 2018), and lending themselves to large-scale analyses of ensembles of proteins. ENM-based studies revealed a close correspondence between the structural changes stabilized upon ligand binding and the intrinsic motions already accessible to the “unbound” protein prior to ligand-binding (Tobi and Bahar 2005; Skjaerven et al. 2011). This led to the concept of *pre-existing paths* of collective structural changes selectively favored upon specific substrate binding (Zheng et al. 2009; Meireles et al. 2011).

In parallel, the evolutionary significance of global modes of motion became clear (Carnevale et al. 2006; Maguid et al. 2006, 2008; Hollup et al. 2011; Bahar et al. 2017). Computations highlighted the coupling between sequence evolution and intrinsic dynamics (Liu and Bahar 2012; Zou et al. 2015; Echave et al. 2016), and experiments demonstrated that the changes in structure (or oligomerization state) stabilized by mutations bear close resemblance to structural changes that accommodate ligand binding (Perica et al. 2014). Evolvability of intrinsic dynamics thus emerged as a major mechanism enabling adaptability to environmental changes, intermolecular interactions, or even mutations (Tokuriki and Tawfik 2009; Haliloglu and Bahar 2015). Recent work further showed that intrinsic dynamics is a major determinant of the impact of missense mutations on function, and that the inclusion of ENM-based features in a machine learning classifier improves the accuracy of pathogenicity predictions (Ponzoni and Bahar 2018).

These observations call for a rigorous evaluation of the conservation/differentiation of structural dynamics in relation to the evolution of sequence and structure in protein families/subfamilies using sufficiently large data sets, and dissecting the contribution of collective motions in different frequency regimes; and the need for a tool to accomplish this task. The present study aims at addressing these needs. We introduce a new interface, *SignDy*, for evaluating the *Sig*nature *Dy*namics of protein families, building on ENM theory and methods implemented in the application programming interface (API) *ProDy* (Bakan et al. 2014). Application to 116 superfamilies of proteins discloses basic principles for functional fitness and diversification: exploiting the robust global dynamics of a versatile fold, and gaining specificity via localized, yet impactful, fluctuations conserved among subfamily members but divergent across subfamilies. We further illustrate the utility of *SignDy* by way of application to three families of folds: 1) leucine transporter (LeuT), 2) periplasmic-binding protein type-1 (PBP-1), and 3) triosephosphate isomerase (TIM) barrel. *SignDy* proves to be an effective tool for quantitative evaluation of both generic dynamics of families, and specific dynamics of subfamilies, identifying the specific modes of motions that distinguish subfamilies (shared by subfamily members but sharply different across subfamilies), and learning how evolutionarily selected folds exploit collective modes of motions in different frequency regimes to reconcile a diversity of sequences and functions with the same architecture.

## New Approaches

The results in this study are generated using a new computing and visualization interface, *SignDy*, designed to enable and automate the evaluation and comparison of the dynamics of structures belonging to evolutionarily related proteins. *SignDy* is built upon the *Pro*tein *Dy*namics (*ProDy*) API (Bakan et al. 2014) launched for *bridging biomolecular structure and function via characterization of dynamics* (Bakan et al. 2011). With more than 100,000 unique visitors and ∼1.7 million downloads (reported by *Google Analytics*), *ProDy* serves as a major resource for exploring a wide range of phenomena, from collective dynamics to sequence coevolution. *SignDy* benefits from 1) theory and methods of ENMs (Bahar et al. 2017; Li et al. 2017), mainly the Gaussian network model (GNM) (Bahar et al. 1997) and the anisotropic network model (ANM) (Atilgan et al. 2001; Eyal et al. 2015); 2) the reconciliation (Chennubhotla and Bahar 2007) of physics-based theories of polymer statistical mechanics and machine learning (ML) algorithms of spectral graph theory; 3) the consolidation of theory and experiments to extract information on motions that facilitate ligand binding, molecular machinery, or allosteric signaling (Tobi and Bahar 2005; Zheng et al. 2009; Fuglebakk et al. 2012; Lopez-Blanco and Chacon 2016); 4) the *Evol* module (Bakan et al. 2014) for evaluating sequence (co)evolution and comparison with structural dynamics (Liu and Bahar 2012); and 5) *NMWiz*, an interactive visualization GUI that interoperates with VMD (Humphrey et al. 1996) and Chimera (Yang et al. 2012).

**Fig. 1.**
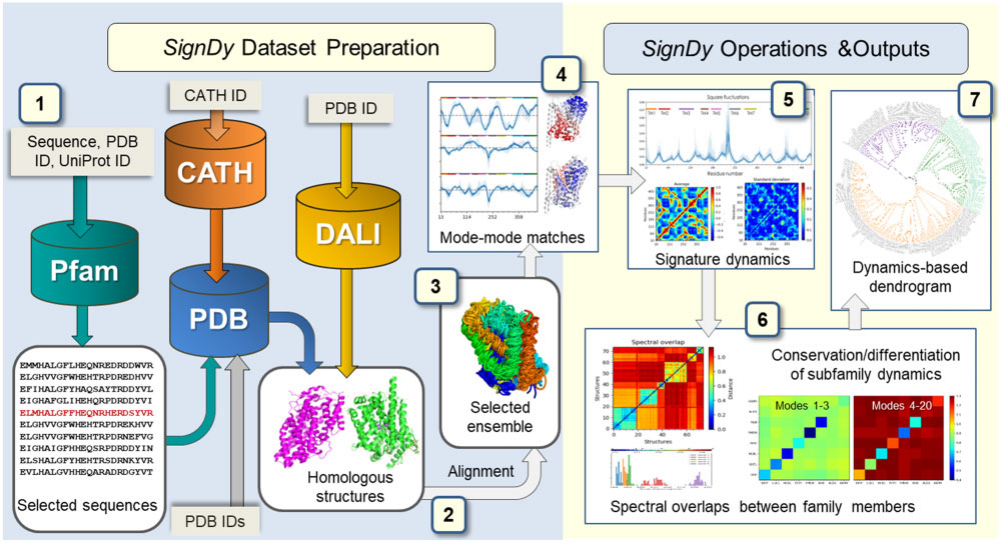
*SignDy* workflow. The workflow is separated into two main parts: data set preparation (*left*; steps 1–3) and *SignDy* operations and outputs (*right*; steps 4–7), described in the text and supplementary methods, Supplementary Material online. *Cylinders* and *light gray rectangular boxes* represent databases and corresponding query inputs, respectively.

*SignDy* is designed as a pipeline composed of seven steps depicted in figure 1, described in the Materials and Methods and the supplementary methods, Supplementary Material online (additional information can be found in online tutorials; http://prody.csb.pitt.edu/signdy/; last accessed April 26, 2019): 1) selection of protein family members to be used as input; 2) structural alignment of members and identification of core residues; 3) refinement of the resulting ensemble and its associated multiple sequence alignment (MSA) based on sequence and structure similarity criteria to select a representative set of homologues; 4) computation of mode spectra using the GNM or ANM, identification of mode–mode matches between family members, and evaluation of the collectivity of modes; 5) characterization of *signature dynamics*, that is, mechanisms of global movements and interresidue cross-correlations shared among family members; 6) quantitative assessment of the conservation/divergence of structural dynamics between family members or subfamilies, broken down into different frequency regimes, and identification of specific modes that unify subfamily members and maximally discriminate between subfamilies, toward gaining insights into the mechanistic basis of functional differentiation of fold families into specific subfamilies; 7) classification of family members based on their dynamics in different frequency regimes, and comparison of the dynamics-based (frequency-dependent) distributions of family members with the distributions based on their sequence, structure, and subfamily function.

We use as metrics of conservation/divergence of structural dynamics among family members the correlation cosines ${\mathrm{cc}}_{k}\left(A,B\right)$ between each matching mode *k* of members *A* and *B*, and the spectral overlap ${\mathrm{SO}}_{\mathrm{ij}}\left(A,B\right)$ for sets of modes (i≤k≤j) in various frequency regimes. Averages over all pairs of members *A* and *B* belonging to specific pairs of subfamilies provide quantitative information on the conservation or differentiation of structural dynamics between subfamilies in different frequency regimes. We analyze the evolution of motions in the global (1≤k≤3), low-frequency (LF; 4≤k≤20), low-to-intermediate frequency (LTIF; 21≤k≤60), and high-frequency (HF; k>60) regimes by assessing which type of motions (global, LF, LTIF, or HF) are shared among family members, how mode collectivity and conservation relate to each other, and which modes accompany, if not control, the differentiation of families into subfamilies.

## Results

### A Unique Signature Dynamics Defined by Conserved Global Motions Characterizes Each Family

**Fig. 2.**
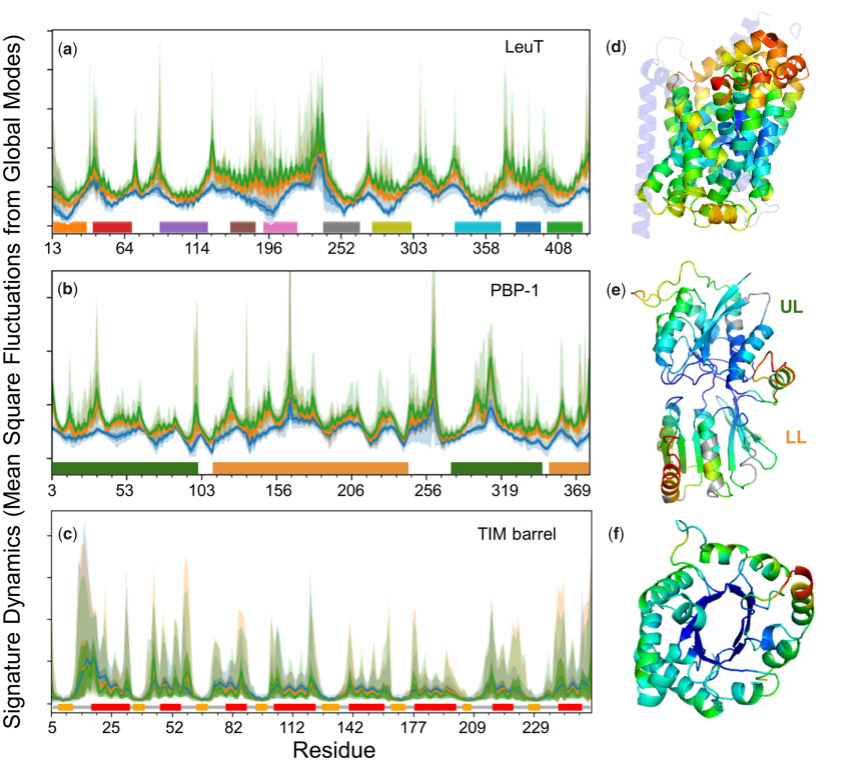
Signature dynamics of each family is robustly defined by global motions uniquely defined by the fold. Panels (*a*–*c*) display the distributions of mean-square fluctuations (MSFs) of residues for the respective fold families LeuT, PBP-1, and TIM barrel. Mobility profiles driven by *k *=* *3 (*blue*), 10 (*orange*), and 20 (*green*) modes are presented, along with their SDs and ranges (bands in *lighter shades*). *Horizontal bars* along the abscissa indicate 1) the transmembrane (TM) helices of LeuT, 2) the upper lobe (UL) and lower lobe (LL) of PBP-1, and 3) the secondary structure (*orange*, β-strands; *red*, α-helices) of TIM barrel. Residue numbers along the abscissa refer to those retained in the ensemble (i.e., sequence positions whose occupancy in the MSA is 0.7 or higher), and deletions are not explicitly shown. (*d–f*) *Ribbon diagrams* of representative members, with core residues color-coded by their mobilities in global modes (1 ≤ *k *≤* *3; *blue*, minimal; *red*, maximal).

Figure 2*a*–*c* illustrates the signature dynamics for three folds, LeuT, PBP-1, and TIM barrel. Information on the corresponding data sets of proteins (Data Sets 1–3) can be found in the respective supplementary tables S1–S3, Supplementary Material online; their sequence, structure, and function distributions are presented in supplementary figure S1, Supplementary Material online. The average mobility profile of residues resulting from global modes of motion (up to *k *=* *3 [*blue*]) and LF motions (up to *k *=* *10 [*orange*] and 20 [*green*] modes) are displayed, along with their standard deviation (SD) and range within each family. Minima and maxima can be traced back to secondary structural elements (indicated by *colored bars* along the abscissa in *a* and *c*) and loops (or disordered regions), respectively. This is due to the high-packing density at secondary structural elements manifested by small-amplitude fluctuations at those regions. The minimal difference between the three curves in each panel indicates the robustness of the signature dynamics defined by global modes. The LF modes in the range 10≤k≤20, which are usually less collective than those in *k *≤* *10, induce increased variations (*shades*) indicative of a differentiation among members while preserving the signature dynamics.

**Fig. 3.**
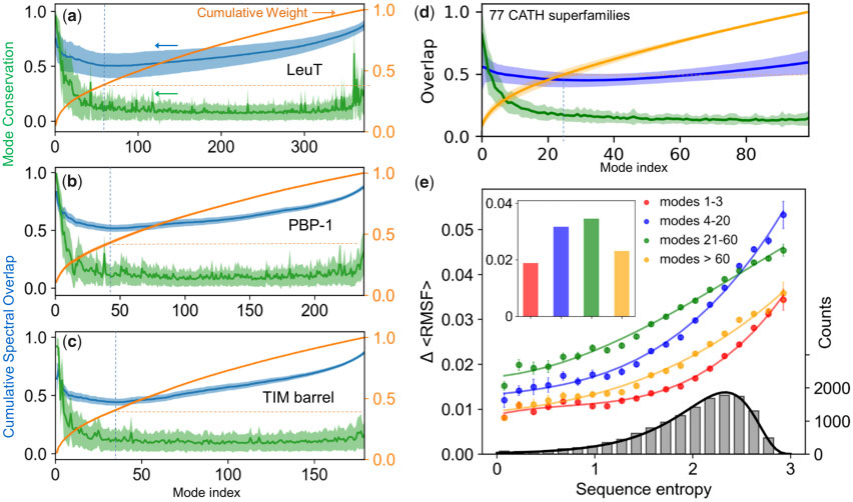
Mode conservation and spectral overlap analysis shows the high conservation of global modes and differentiation of LTIF modes among (super)family members. (*a*–*c*) Mode conservation profile given by mode–mode correlation cosines $\langle {\mathrm{cc}}_{k}\rangle$ averaged over all family members (*green*), cumulative spectral overlaps (*blue*), and cumulative weights of individual modes (*orange*) plotted as a function of mode index for LeuT, PBP-1, and TIM barrel folds, respectively. The curves display the averages over all members in each family and the bands show the SDs. In all three cases, the mode conservation decreases sharply from 0.96 ± 0.03 for *mode 1*, to 0.63 ± 0.23 for *mode 5*, and 0.18 ± 0.15 for mode 30. *Dashed vertical blue lines* indicate the region where the cumulative spectral overlap is minimal, and *dashed orange horizontal lines* indicate the corresponding cumulative weight. (*d*) Same result for first 100 modes obtained for 77 CATH superfamilies with *N *>* *100 (see supplementary table S4, Supplementary Material online). The range 1 ≤ *k *≤* *100 covers four regimes of motions: global/softest (*k *≤* *3), LF (4 ≤ *k *≤* *20), LTIF (21 ≤ *k *≤* *60), and HF (*k *≥* *60). (*e*) Change in root-mean-square fluctuations, ΔRMSF, computed for all residues in each of the 77 CATH superfamilies as a function of sequence variations (sequence entropy) evaluated for four frequency regimes (*labeled*). The corresponding average values are shown by colored bars in the inset. The colored curves are weighted least square fits to computed data using cubic regression, with respective correlation coefficients >0.99. The distribution of sequence entropy for the 77 superfamilies, shown by the *gray* bars (*right ordinate*) with a bin size of 0.15 and an average value is 2.0, fits a lognormal probability distribution (*black curve*) with a correlation coefficient of 0.997.

To assess the level of conservation of global modes within families, we evaluated the mode–mode correlation cosines $\langle {\mathrm{cc}}_{k}\rangle$ averaged over all family members for each equivalent mode *k*. The results are presented in figure 3*a*–*c* (*green curve*s and *shades* for the respective averages and SDs). Sharp peaks at the lowest frequency end of the spectra and rapid decays with increasing mode number confirm the conservation of global modes.

### Robust Global Modes Define Signature Dynamics

To confirm the dominance of global modes as a determinant of family signature dynamics, we examined their level of conservation within CATH superfamilies. To this aim, we considered 116 highly populated superfamilies (Data Set 4) which overall include 26,899 Protein Data Bank (PDB) structures (supplementary table S4 and fig. S2, Supplementary Material online). For each superfamily, we computed the mode–mode correlation cosine curves, and then evaluated the average over all superfamilies. The resulting master curve and its SD (shown in fig. 3*d*, *green curve* and *shade* for 1≤k<100) consistently show that global modes are highly conserved. The average correlation cosine for the top-ranking mode (*k *=* *1) of superfamily members is 0.80 ± 0.19 and drops to 0.20 ± 0.07 for *k *=* *20 (supplementary fig. S3*a* and *b*, Supplementary Material online). Higher modes display a plateau with minimal (0.1–0.2) correlation.

Larger proteins/domains have access to a broader conformational space and a wider spectrum of motions. One might expect their dynamics to be more heterogeneous, leading to weaker mode conservation among members. Computations (supplementary fig. S3, Supplementary Material online) showed, however, that the dependency of mode conservation propensity on protein size is minimal. The top-ranking modes exhibit strong correlations, irrespective of the size of the protein, again confirming that a handful of global modes robustly define the signature dynamics of the family.

### Motions in the LTIF Regime Differentiate the Dynamics of Family Members

Figure 3*a*–*c* illustrates the spectral overlaps (*blue curves*) for the three example folds. In each case, the cumulative spectral overlap $\langle SO_{1n}\rangle$ is plotted as a function of the total number of modes, *n*, included in the analysis, together with the corresponding variation among family members (*lighter blue band*). The curves reflect two counter effects: first, there is a peak at the lowest frequency end, consistent with the conservation of global modes. The overlap rapidly decreases with increasing *n*, due to the dissimilarity of the newly added modes. This differentiation between family members is consistent with the rapid drop in mode conservation (*green curve*) shown in figure 3*a*–*c* for LeuT, PBP-1, TIM barrel families as well as that for CATH superfamilies (fig. 3*d*). Then, a new regime is observed, the *LTIF* regime, which includes modes 20–60 approximately, where the spectral overlap is minimized (minima indicated by *dashed vertical lines*). Finally, an opposite effect takes over, manifested by an increase in overlap. This arises from the increased coverage of the space of conformational changes (shown in the *orange curve*), consistent with the theoretical limit of $SO_{1n}\left(A,B\right)\to 1$ as the complete space of motions is considered. The minimum in $\langle SO_{1n}\rangle$ occurs for n≤50.

The LTIF regime where the cumulative spectral overlap is minimized emerges as a determinant of the specificity of family members. The percent contribution of the modes in this regime to the overall spectrum amounts to ∼25% (see the increase in the cumulative weight of modes [*orange curves* in fig. 3*a*–*c*] in this interval), which means a substantial contribution to alter dynamics, while retaining the generic behavior.

Further calculations performed for CATH superfamilies (fig. 3*d*) corroborated the same trends. Supplementary table S4, Supplementary Material online, lists the spectral overlap calculated for *n *=* *3, 20 and all (*N*−1) modes for each superfamily, along with their SDs, and supplementary figure S3*c*, Supplementary Material online, displays their histogram. The spectral overlap achieved by global modes, $\langle SO_{1-3}\rangle$, averaged over all superfamilies, is 0.55 ± 0.25, despite the low (<0.10) cumulative weight of this small set of modes. The addition of modes in the LF regime lowers the cumulative overlap to 0.45 ± 0.15, even though a larger subspace of conformational changes is sampled, indicating the dissimilarities in conformational motions among members in this regime. A high overlap ($\langle SO_{\mathrm{all}}\rangle$ = 0.84 ± 0.02) is recovered by the ensemble of all modes, which, by definition, forms a complete basis set that spans all possible conformational changes.

Overall, these data underscore the role of motions in the LTIF regime in differentiating family members within a given fold family, which will be further elaborated below.

### Increased Sequence Heterogeneity in a Given Fold Family Manifests Itself by Higher Differentiation of Dynamics, Especially in the LTIF Regime

Our earlier work showed that sequentially conserved sites are also distinguished by their restricted fluctuations; or the mobility of residues, reflected by their mean square fluctuations (MSFs) around their mean positions, increases with increasing Shannon entropy (*H*) at the corresponding sequence position (Liu and Bahar 2012). That study established the correlation between sequence variation and *conformational* flexibility (RMSF). Here, we investigated one further property, the *change* in flexibility, ΔRMSF, at a given position among family members, which is a metric of the extent of differentiation in the equilibrium dynamics between family members.

To this aim, we first evaluated the level of sequence heterogeneity within each family, using Shannon entropy as a metric. The resulting distribution among 13,648 residues belonging to 77 CATH families (after excluding the small folds with *N *<* *100 residues) is shown by the histogram (*gray bars*) in figure 3*e*. The histogram perfectly fits a lognormal distribution in support of the accurate sampling of sequence variabilities by the examined set (supplementary table S4, Supplementary Material online). The changes in residue fluctuations, $\Delta \langle \mathrm{RMSF}\rangle$ (where the triangular brackets indicate the averages over residues with sequence entropy in the bin corresponding to the bar underneath), exhibit a smooth increase with increasing sequence entropy (*four curves* in fig. 3*e*), confirming that sequentially diverse families exhibit higher differentiation in their dynamics.

The results are presented for different subsets of modes: global (k≤3), LF (4≤k≤20), LTIF (21≤k≤60), and HF (k≥60) regimes. The *bar plot* in the inset displays the $\Delta \langle \mathrm{RMSF}\rangle$ averaged over all sequence entropies for the four respective groups. These results clearly show the dominant role of LTIF motions in imparting the member-specific differences in the fluctuation spectrum of individual family members, except for the high-sequence entropy region. In this case, differentiation of the modes shifts toward slower modes, as can be seen from the crossover between the LF and LTIF curves. The shift to LF modes reflects the earlier divergence of modes along the mode spectrum, in tandem with the higher divergence of sequence.

A closer examination shows that $\Delta \langle \mathrm{RMSF}\rangle$ contributed by the global modes is relatively flat with respect to sequence entropy in the range H≤1.5. This insensitivity to sequence variations suggests that global dynamics are more conserved compared with sequence, presumably consistent with the slower divergence of structure, compared with sequence. Supplementary figure S3*d*, Supplementary Material online, further shows that diverging structures encode diverging dynamics despite the rather narrow root-mean-square deviation (RMSD) range. This dependency is stronger when all modes (*red dots*) are considered, as opposed to global modes *(orange dots*), confirming the increased differentiation of mode spectra with addition of higher modes. There is, however, some variation of spectral overlap with sequence identity (supplementary fig. S3*e*, Supplementary Material online), indicating that diverging sequences encode diverging dynamics too, which will become even clearer by focusing on subfamily dynamics next.

### Differentiation of Protein Families into Specific Subfamilies Is Accompanied by the Evolution of LTIF Motions

Consider a family composed of *m* subfamilies (or a superfamily of *m* families). For example, the currently considered TIM family contains eight subfamilies (with at least four members). Subfamily classification is based on the specific functions of family members, for example, in the case of TIM barrel, we have aldolases class 1 (ALD1), glycosidases (GLYC), and phosphenolpyruvate binding domains (PEPE). Of interest is to assess to what extent subfamily members share similar modes among themselves, and to what extent they differ from other subfamily members. In other words, is the differentiation of fold families into specific subfamilies accompanied, if not driven, by a subset of modes that typifies the subfamily, and distinguishes it from all other subfamilies?

Note that subfamily members are not necessarily sequentially close or structurally close, but they belong to the same subfamily because of their shared biological (e.g., specific enzymatic) activities. In this respect, it is of interest to see if their common functions are supported by common mechanisms of action, or shared modes. Another way of asking the same question is which particular modes, or modes in which frequency regime, unify members *within* subfamilies, while ensuring maximal differentiation *between* subfamilies themselves. Toward this goal, we evaluated the spectral distances ${\langle d_{\mathrm{ij}}\rangle }_{m_{p},m_{s}}$ between subfamilies *p* and *s*, composed of *m_p_* and *m_s_* members, respectively, based on the similarity of their modes i≤k≤j (see Materials and Methods and Supplementary Material online).

**Fig. 4.**
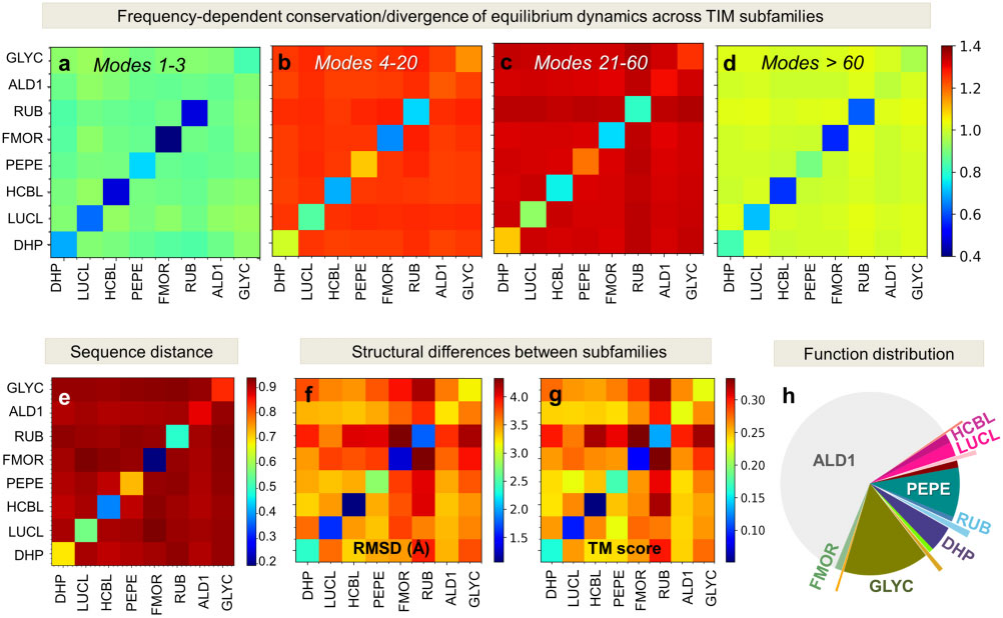
Low-to-intermediate frequency modes discriminate between subfamilies with different functions belonging to the TIM barrel fold family. (*a–d*) Subfamily–subfamily distance matrices based on structural dynamics, evaluated for eight TIM subfamilies. Subfamily acronyms are listed along the axes (see full names in supplementary table S3, Supplementary Material online, and their distribution in supplementary fig. S1*c*, Supplementary Material online). Spectral distances ${\langle d_{\mathrm{ij}}\rangle }_{m_{p},m_{s}}$ averaged over all *m_p_* and *m_s_* members of respective subfamilies (see supplementary methods, Supplementary Material online) are shown by color-coded elements (*red*: long; *blue*: short; see the bar on the *right*). Results are displayed for four frequency regimes, global, LF, LTIF, and HF, in the respective panels (*a*–*d*), as indicated by the ranges *i* ≤ *mode* ≤ *j*. Diagonal terms show the average distances between members *within* subfamilies based on the motions in the particular frequency window; and the off-diagonal terms show those *across* subfamilies. The LTIF regime (modes 21–60) provides the sharpest discrimination between subfamilies; whereas modes in both the global (*a*) and HF (*d*) regimes are relatively conserved. For comparison, we present the sequence distances (*e*) and structural distances (*f* and *g*, using RMSD and TM-score as metrics) between subfamilies. Note that the subfamily–subfamily spectral distances in the LTIF regime (panel *c*) conform closely to their functional classification (panel *h*) defined by CATH, rather than their structural similarities (panels *f* and *g*), in strong support of the significance of LTIF motions in the evolution of function.

Figure 4*a*–*d* and supplementary figure S4*a*–*d*, Supplementary Material online, illustrate the respective results for TIM and PBP-1 families. Results are presented for the global, LF, LTIF and HF frequency regimes (respective panels *a*–*d*) by color-coded matrices. Diagonal elements describe the level of conservation of dynamics within subfamilies; whereas off-diagonal terms represent the distances between pairs of subfamilies, with *dark red* entries indicating a strong divergence. We note that the LTIF modes are maximally distinctive across families, followed by LF modes, while the global modes and, interestingly, HF modes retain similarities. The strong discrimination provided by the LTIF regime between subfamilies—a feature apparent in the large-scale examination of CATH superfamilies, is now clearer with the subfamily–subfamily distance maps based on subfamily dynamics.

Further comparison of the conservation/divergence of structural dynamics across subfamilies with their sequence and structure similarities (panels *e*–*g* in fig. 4 and supplementary fig. S4, Supplementary Material online) reveals that the correlations (or lack thereof) between the mode spectra of subfamilies in different regimes closely parallel sequence properties, rather than structural similarities/dissimilarities. The latter was assessed by two metrics, average RMSD between subfamilies and average Template Modeling score (TM-score) (Zhang and Skolnick 2004), which yielded almost identical results. In other words, the division of families into subfamilies relates to the differentiation of their dynamics, more than the differentiation of their structure, in support of the direct relevance of motions/dynamics to subfamily function. Overall these results demonstrate that the specific mechanisms that distinguish subfamilies can be traced back to the intrinsic modes in the LTIF regime.

### Evolutionary Conservation of Modes Shows a Unique Dependency on Their Collectivity

**Fig. 5.**
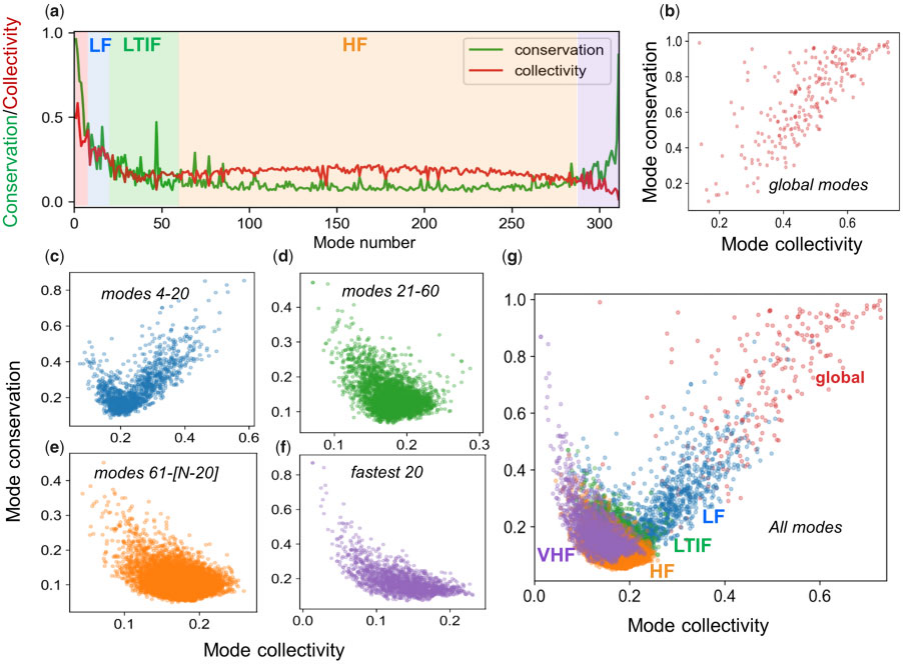
Correlation between mode conservation and mode collectivity. (*a*) Comparison of the conservation (*green*) and collectivity (*red*) profiles of all modes, illustrated for 3′5′-cyclic nucleotide phosphodiesterase, catalytic domain (CATH id: 1.10.1300.10). The different frequency regimes of the mode spectrum are indicated by semitransparent color-coded shades (*red*: global; *blue*: LF; *green*: LTIF, *orange*: HF and *violet*: VHF). (*b–f*). Mode conservation versus mode collectivity scatter plots for the GNM mode spectra of 15,636 proteins belonging to 77 CATH superfamilies, in five different frequency regimes, as labeled. Panel (*g*) displays all modes (from *b* to *f*) on the same plot, with the abscissa and ordinate representing the collectivity and conservation, respectively, of the modes in each family.

Global modes are usually known to be highly collective, that is, they cooperatively embody large portions of the structure. HF modes, on the other hand, are highly localized. In order to understand whether the conserved and not conserved modes in the same frequency range are characterized by different levels of collectivity, we compared the conservation profile of the modes and their collectivity profile observed in superfamilies. The results are illustrated for an example CATH superfamily (3′5′-cyclic nucleotide phosphodiesterase catalytic domain) in figure 5*a*, and similar results are shown for a series of CATH superfamilies in supplementary figure S5, Supplementary Material online. In each case, the *green curve* displays the conservation profile ($\langle {\mathrm{cc}}_{k}\rangle$ similar to fig. 3*a*–*c*) and the *red curve* the collectivity profile ($\kappa_{k}$) for all modes (1≤k≤N-1) obtained by the GNM. All the curves practically show the same trend: a positive correlation between conservation and collectivity in the global and LF regimes, followed by the negative correlation in the LTIF and HF regimes, and strikingly an increase in conservation, accompanied by a decrease in collectivity at the fastest end of frequency spectrum, designated here as the very high-frequency (VHF) regime, already discerned in figure 3*a*–*c*.

Systematic analysis of all 77 CATH superfamilies led to the plots in figure 5 (panels *b*–*f*). In the case of global modes, the more collective modes are also those that tend to be evolutionarily conserved (panel *b*), and the same trend can also be seen in LF modes (in panel *c*) although we can detect some modes that exhibit the opposite behavior, that is, they exhibit high conservation despite having low collectivity. This type of anticorrelation dominates the rest of the spectrum, including the LTIF, HF, and VHF modes (panels *d*–*f*). Panel (*g*) displays all the results, thus allowing us to clearly view the complex relationship between collectivity and conservation, broken down into different regimes.

### Conserved Local Motions Specific to Subfamilies Can Be Detected among HF Modes

It is interesting to observe peaks at relatively higher modes in the mode conservation curves (figs. 3*a*–*c*and 5*a* and supplementary fig. S5, *green curves*, Supplementary Material online). These signal the conservation of local events among subsets of members. Figure 4*d* and supplementary figure S4*d*, Supplementary Material online, further support the conservation of HF modes within subfamilies, and even across subfamilies. Early applications of the GNM pointed to evolutionarily conserved sites distinguished by HF modes relevant to stability, even though the high sensitivity of HF modes to structural details would preclude us from generalization (Bahar et al. 1998). The consolidation of such modes over all family members by *SignDy* provides a framework for identifying such critical sites, illustrated in supplementary figure S6, Supplementary Material online for the TIM barrel and PBP-1 families, which may assist in assessing the pathogenicity of single amino acid variants (SAVs) (Ponzoni and Bahar 2018).

### Dynamics-Based Dendrograms Distinguish between Substates and Subfamilies of Structural Homologs

**Fig. 6.**
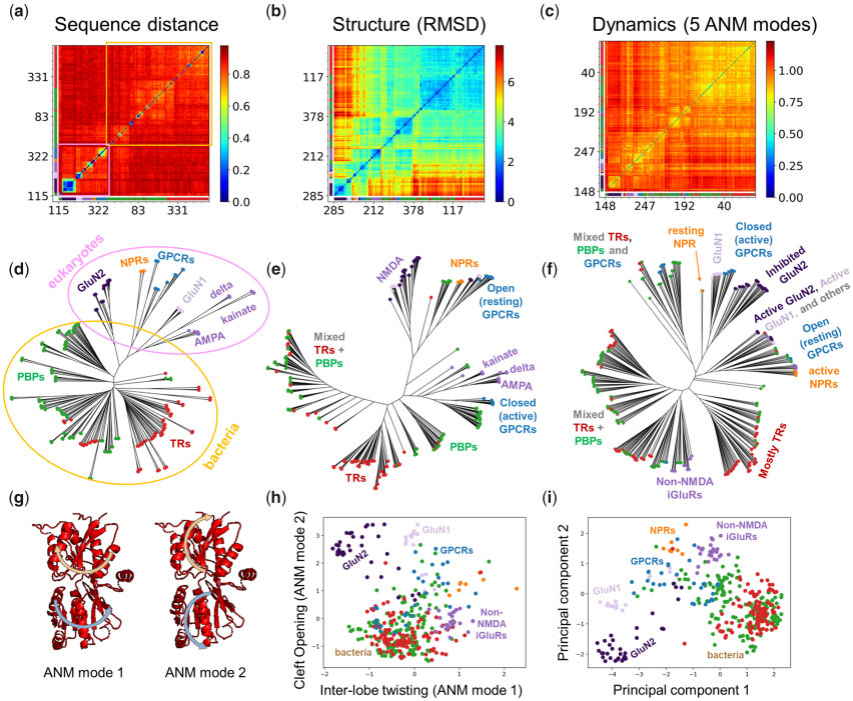
Categorization of family members based on their sequence, structure, and dynamics. (*a–f*) Distance matrices (*a–c*) and corresponding dendrograms (*d–f*) for PBP-1 family members based on (*a* and *d*) Hamming distance between sequences, (*b* and *e*) RMSD between structures and (*c* and *f*) spectral distance between global ANM modes (*k *=* *5). The numbers and colors along the axes correspond to the order of the conformers based on RMSD clustering and the subfamilies to which they belong (see supplementary table S2, Supplementary Material online). In the trees, each node represents a member and the colors and labels correspond to subfamilies along with conformational/functional states. In the sequence case (*a* and *d*), there is a clear distinction between bacteria and eukaryotes, highlighted in *blue* and *orange*, respectively. (*g*) The first two global signature ANM modes are shown with arrows illustrating the opposite motions of the upper and lower lobes. Mode 1 (*left*) shows a twisting/untwisting motion and mode 2 (*right*) shows an opening/closing motion as shown in supplementary movies 1 and 2, Supplementary Material online. (*h*) Projection of family members onto a 2D space spanned by the first two signature ANM modes clusters family members based on global mode spectra akin to panels (*c*) and (*f*). (*i*) Projection of family members onto a 2D space spanned by the first two principal components of structural variation clusters family members akin to panels (*b*) and (*e*).

Using sequence-, structure-, and dynamics-based distance metrics, we generated the maps and dendrograms presented in figure 6 and supplementary figure S7, Supplementary Material online, for the PBP-1 family, named after periplasmic binding proteins (PBPs) in bacteria that capture solute in the periplasm and supply them to ABC transporters (Quiocho and Ledvina 1996). This fold has been used in a number of other proteins, where it is involved in signal transduction in a variety of eukaryotic multidomain receptors (Felder et al. 1999) as well as bacterial transcription regulators (TRs) such as LacI (Swint-Kruse and Matthews 2009). The maps in figure 6 show the pairwise distances between the sequences (*a*), structures (*b*), and dynamics (*c*) of the family members, which are used for constructing the dendrograms (*d*–*f*) under each map. They are colored from most similar in *dark blue* to most different in *dark red*. The members are reordered along the axis based on the dendrograms to aid with visualization and the numbering of family members along the axes corresponds to that in supplementary table S2, Supplementary Material online, which is based on the structure dendrogram. The color-code along the two axes refers to function annotations in supplementary figure S1*b* and table S2, Supplementary Material online.

At the sequence level (fig. 6*a* and *d*), we observe a clear separation between bacterial and eukaryotic family members (highlighted in *orange* and *pink*, respectively). Smaller clusters with higher sequence similarity within these two groups (*yellow*, *green*, and *blue* boxes in the fig. 6*a*) correspond to functional groups such as different iGluRs (AMPA, kainate, delta, and NMDA receptors), class C G-protein-coupled receptors (GPCRs), and natriuretic peptide receptors (NPRs). The structure-based dendrogram (fig. 6*e*) reveals more heterogeneity including a splitting of the bacterial, GPCR, and NPR structures into open and closed forms but performs less well at distinguishing subfamilies.

Dynamics-based classification based on global ANM or GNM modes (fig. 6*f* and supplementary fig. S7*a*, Supplementary Material online, respectively) enables us to discriminate between active and inactive states, distinguished by conserved opening/closing and twisting motions (Krieger et al. 2015) driven by the two signature ANM modes (fig. 6*g* and supplementary movies 1 and 2, Supplementary Material online). This also results in a mixing of bacterial and eukaryotic proteins, because active (or inactive) states of bacterial PBPs rather resemble their eukaryotic counterparts in the same state. This can be seen more clearly in panel *h* where we project the classification onto these two signature ANM modes, which may be compared with the structural classification (panel *i*) based on the principal component analysis of the structures. Dissecting of mode spectra into different frequency regimes (supplementary fig. S7*b*–*d*, Supplementary Material online) provides better classification of subfamilies. Especially the dendrograms based on LTIF modes 21–60 (supplementary fig. S7*c*, Supplementary Material online) provide a clear separation of subfamilies, consistent with supplementary figure S4, Supplementary Material online.

### Application to LeuT Fold Family Shows How Signature Dynamics Favors Functional and/or Multimerization Mechanisms

The LeuT fold, first resolved for a bacterial leucine transporter (Yamashita et al. 2005), is shared by many prokaryotic and eukaryotic secondary transporters despite their low-sequence identity (Shi 2013; Drew and Boudker 2016). It is composed of 12 TM helices that alternate between outward-facing (OF) and inward-facing (IF) conformations during the transport cycle. The former favors the uptake of substrate from the extracellular (EC) region, and the latter its release to the intracellular (IC) region, accompanied by the cotransport of Na^+^ ions, and in some cases by the antiport of other substrates/ions (Kazmier et al. 2017). Family members include dopamine transporter (DAT), multihydrophobic amino acid transporter (MhsT), benzyl-hydantoin transporter (Mhp1), sodium/galactose transporter (vSGLT), glycine betaine transporter (BetP), carnitine/butyrobetaine antiporter (CaiT), and arginine/agmatine antiporter (AdiC). See supplementary table S1 and figure S1*a*, Supplementary Material online, for sequence and structure properties of the 85 members studied here, figures 2*a*and 3*a* for signature dynamics, mode conservation, and spectral overlap between family members.

**Fig. 7.**
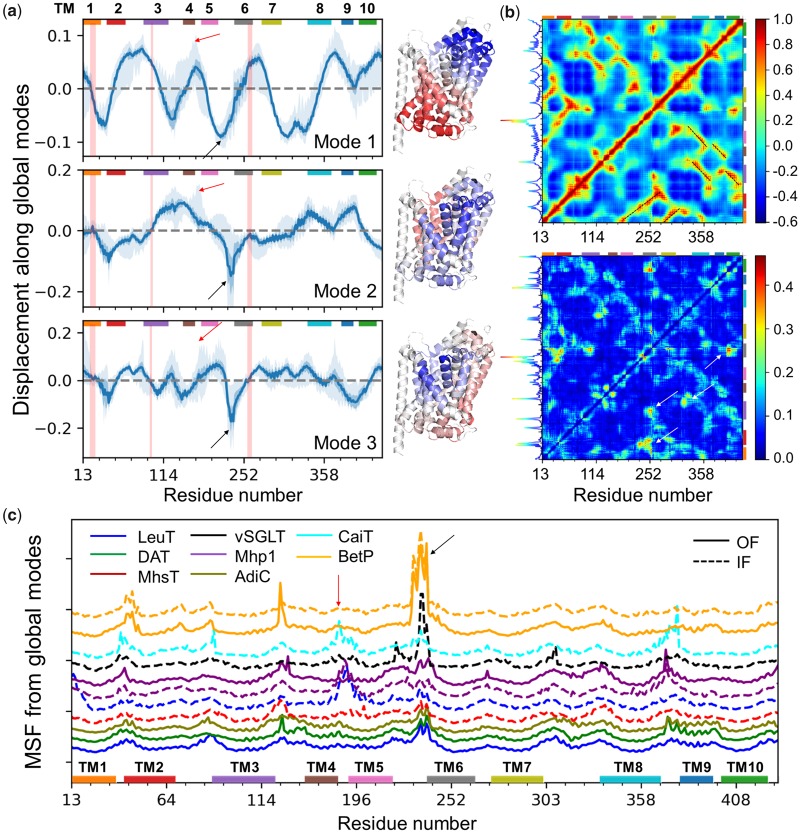
Generic and specific features of LeuT fold dynamics. (*a*) Global mode shapes and displacements along the global modes shared by family members (mean profiles, *solid curves*), and their differentiation (SDs; *darker shaded area*), and the full range of variations (*lighter shaded area*). *Colored bars* along the upper abscissa indicate the TM domains. *Pink vertical bands* indicate the residues lining the substrate-binding pocket, which show minimal spatial displacements. *Red* and *black arrows* indicate the locations of IL2 and EC3/H7, respectively, the high flexibility of which is essential to substrate recognition and multimerization. The *ribbon diagrams* generated for a representative LeuT structure (PDB ID: 2A65) are color-coded (from *blue* to *red*) by the size and direction of motions (from negative to positive) in each mode. (*b*) Generic covariance map (*top*) and its SD (*bottom*), based on *k *≤* *20 modes. See more details in supplementary figure S10, Supplementary Material online. Specific residue pairs whose cross-correlations significantly depart from the generic covariance are indicated by *white arrows* (*bottom*). The curve along the left ordinate shows the row-average. The peak at TM6 suggests a driving role in eliciting cooperative changes. (*c*) Detailed view of the global/soft motions (*k *≤* *5) for 13 representative structures from 8 transporter families (*labeled*), in inward-facing (IF; *dashed*) and/or outward-facing (OF; *solid*) states. Differences in peaks/minima reveal member-specific features, for example, IL2 fluctuations (*red arrow*) are prominent in the IF states of LeuT, Mhp1, and CaiT, but not in the IF state of MhsTs, BetP, and vSGLT nor the OF states; EL3/H7 (*black arrow*) motions are suppressed in most IF conformers except in vSGLT, BetP, and to some extent CaiT, where this specific region facilitates trimerization (see also supplementary fig. S9d, Supplementary Material online). The curves are vertically shifted for visual clarity.

Here, we focus on transport and multimerization mechanisms of LeuT members. Figure 7 and supplementary movies 3–5, Supplementary Material online, reveal how the three global modes operate in a complementary way to enable substrate transport: they divide the fold into two parts from three orthogonal perspectives, resulting each in concerted opposite-direction (anticorrelated) fluctuations (or breathing motions) of the respective parts. Their combination allows for the cooperative opening and closing of the central substrate/ion-binding pocket (supplementary fig. S8, Supplementary Material online). The close-to-zero values in figure 7*a* (indicated by *vertical pink shades*) indicate pivotal sites at the interface between oppositely moving substructures.

Closer examination reveals large displacements in EC loop 3 (EL3; known as helix H7 in BetP and CaiT) (*black arrows* in fig. 7*a*). The transporters exhibit large structural heterogeneities at this region (supplementary fig. S9*a*, Supplementary Material online). However, the movement of EL3 is not random. On the contrary, it is driven by a cooperative mode (ANM mode 2) that enables the transition between OF and IF states of the transporter; and further motion of BetP H7 along the same direction/mode allows for intersubunit contacts that stabilize the trimer (Ponzoni et al. 2018) (supplementary fig. S9*c* and movie 6, Supplementary Material online). H7 also takes part in the trimerization interface of CaiT (supplementary fig. S9*d*, Supplementary Material online).

Another region distinguished by its conformational adaptability is IC loop 2 (IL2; *red arrow* in fig. 7*a* and *c*). This region undergoes large rearrangements during the OF ↔ IF transitions of LeuT (Krishnamurthy and Gouaux 2012), Mhp1 (Shimamura et al. 2010), MhsT (Malinauskaite et al. 2014), and BetP (Perez et al. 2012), the directions and the sizes of the deformations varying between members. The departure from the generic signature profile at this region suggests a role in imparting specificity (see also fig. 7*c*). Finally, the cross-correlation maps (fig. 7*b* and supplementary fig. S10, Supplementary Material online) highlight the structural elements that undergo coupled same-sense (*red*) or opposite-sense (or anticorrelated; *blue*) motions. The largest variations in cross-correlations (lower map in fig. 7*b*) take place in the motions of TM6 with respect to TMs 1–3 and 10. These interhelical distances have been noted to define the extent of opening/closure of the EC and IC vestibules (Cheng and Bahar 2014; Drew and Boudker 2016). TM1 movements are shown here to be anticorrelated with respect to TM10 which forms a coherent block with TM5 and TM7. These observations are consistent with recent H/D exchange mass spectrometry experiments where partial unwinding of TM1, 5, 6, and 7 drives the OF → IF transition (Merkle et al. 2018).

## Discussion

In recent years, there has been an increasing interest in interpreting sequence evolutionary trends in the light of biophysical models, reconciling evolutionary biology, and structural biophysics (Liberles et al. 2012; Echave and Wilke 2017). Structural stability and related functions such as residue packing density are key constraints in sequence conservation and evolutionary change rate (Echave et al. 2016). Yet, stability alone is not sufficient for functionality. Many proteins achieve their function by virtue of their conformational flexibility (Zheng et al. 2009; Skjaerven et al. 2011; Haliloglu and Bahar 2015). While the conservation of *sequence*, or sequence evolution rate, closely relate to structural stability and thermodynamics, the conservation of *structure* and its evolution might be closely determined by its adaptability to functional requirements. The present study aimed at shedding light to the relation between biomolecular dynamics and evolution of structure and function. We examined subfamilies from the perspective of their structural dynamics and identified which frequency windows of the mode spectrum naturally provide the most discriminative description of subfamilies, that is, which modes entail motions shared among subfamily members but sharply divergent between subfamilies. Decomposition of the mode spectrum into the contribution of different frequency windows unambiguously revealed the evolutionary significance of a well-defined subset of modes, those lying in the LTIF regime. These modes endow subfamily members with subfamily-specific motions, or mechanisms of action, and they provide maximal discrimination between subfamilies in accord with their functional categorization in the CATH database.

Pioneering studies that introduced the concept of evolution of structural dynamics and/or its relation to sequence evolution traditionally focused on experimental data, for example, α-carbon fluctuations (B-factors) (Maguid et al. 2006, 2008), the coupling between sequence variability and structural dynamics (Liu and Bahar 2012; Nevin Gerek et al. 2013), or diversity of conformers resolved for well-studied proteins in the PDB (Juritz et al. 2013). The present study, inspired by earlier observations and motivated by the need to gain a deeper understanding of the principles that control the conservation/divergence of structural dynamics led to design and implementation of a new interface, *SignDy*. *SignDy* permitted us to systematically analyze 15,636 proteins in 77 CATH superfamilies, and revealed features that could not be discerned if it were not for serial analysis of large ensembles of CATH superfamilies. We discerned for the first time the differences in the conservation of modes in different frequency regimes, and the close relationship between the dissimilarities in the LTIF modes and the structural variations and specific mechanisms of action that distinguish subfamilies.

### Distinctive Evolution of Modes in Different Frequency Regimes and Relation to Differentiation into Subfamilies

We have conducted a thorough examination of the evolution of structural dynamics by focusing on four windows of mode spectra: global modes (*k *=* *1–3), slow (low frequency, LF) modes (*k *=* *4–20), LTIF modes (*k *=* *21–60), and fast (high frequency, HF) modes (*k *>* *60). These ranges are estimated from the average behavior of 77 CATH superfamilies, and the boundaries between these regimes may vary slightly among different protein families. Notably, different frequency regimes exhibited different relationships to the evolution of structure and function. The global modes are highly conserved across all members of the family, that is, they are resilient to change throughout evolution, presumably due to their role in defining the signature dynamics of the family. The LF regime, on the other hand, exhibits a dependency on the type of subfamily, thus underlying the differentiation of subfamily members in terms of their dynamics. This effect is further pronounced in the LTIF regime. The LTIF regime ensures maximal discrimination between the dynamics, or accessible mechanisms of action, of subfamily members, while also accomplishing the highest similarity among members within subfamilies. Major contribution to the specificity of subfamilies originates in the LTIF regime. Finally, the HF regime makes little contribution to structural divergence (fig. 3*e*). Yet, the same regime has several “conserved” modes, similar to the global modes, but completely different in terms of their collectivity (see figs. 3*a*–*c* and 5 and supplementary figs. S5 and S6, Supplementary Material online). While HF motions are usually viewed as noise in molecular simulations, the current approach that yields an analytical solution (unique to each fold) reveals the evolutionary conservation of selected HF fluctuations among family members, across all subfamilies. In sharp contrast to global modes, HF modes are highly localized, but presumably important enough to biological function such that they are retained across subfamilies throughout evolution of sequence and structure. These findings link biomolecular structural dynamics (topology-encoded collective modes of motion) to the evolution of structure and function.

### New Insights by Serial Examination of Large Ensembles of Protein Families

*SignDy* differs from existing computational methods for exploring structure-based dynamics and its evolution in several ways: first, it is fundamentally different from full atomic models and simulations, which do not lend themselves to systematic comparative analysis of hundreds, if not thousands, of proteins’ dynamics, even with technological advances that permit to up to milliseconds dynamics of small proteins (Dror et al. 2012). Second, at the heart of the methodology is the prediction of structural dynamics and in particular, the global modes of motions and correlations by a model (ENM) that lends itself to analytical solutions. The adoption of such a method that dissects structural dynamics was essential to distinguishing conserved and divergent motions of families and superfamilies.

Many ENM-based predictive studies of comparative dynamics provided valuable insights on the evolution of motions in selected systems (Carnevale et al. 2006; Micheletti 2013; Dutta et al. 2015; Zou et al. 2015; Tiwari and Reuter 2016, 2018; Ponzoni et al. 2018). Other comparative studies highlighted the bridge between structural dynamics and sequence evolution (Liu and Bahar 2012; Nevin Gerek et al. 2013). Significant efforts have been deployed for developing interfaces that enable principal component analysis of structurally known sequence homologues, comparisons with ANM predictions (Bakan et al. 2011; Skjaerven et al. 2014), and comparison with sequence coevolution properties (Bakan et al. 2014). More recently, Reuter and coworkers (Tiwari and Reuter 2016) performed an insightful ENM analysis of 23 proteins belonging to five different families that share the TIM barrel fold, to highlight the adaptability of the fold to various functions by virtue of its intrinsic signature dynamics. However, a large-scale systematic study of superfamilies of protein folds that share very low-sequence identity and accommodate a diversity of functions has been a challenge due to many obstacles, starting from the selection/retrieval of (super)family members (which cannot be done by PDB BLAST search due to low-sequence identity), and the optimal structural alignment of members. *SignDy* provides automated tools that surmounts these obstacles and allows for comparing the dynamics of CATH superfamily members that share similar structures but minimal sequence identity and a broad range of functional diversity. Another strength of *SignDy* is the use of GNM (in addition to ANM), which has been shown in numerous applications to yield results in better agreement with experiments than ANM (Bahar et al. 2017). The tool highlights features that could not be unambiguously detected upon examination of individual cases, such as the trade-off between adaptability and specificity as discussed next.

### Compromise between Adaptability and Specificity

It is well known that sequence diverges much faster than structure. In other words, the sequence space is much larger than the structure/fold space. The mapping of various sequences into a small number of folds, or a relatively small set of fold superfamilies (e.g., ∼100 examined here that cover almost ¼ of PDB structures), does not, however, prevent proteins from achieving a broad diversity of functions. The latter is enabled by conformational dynamics.

The present study suggests that conformational dynamics supports the selection of folds in two ways: first, all family members share the fold-encoded global modes, or signature dynamics, that presumably underlie the versatility of the fold, for example, the different members may exhibit different levels of interdomain opening, or global twisting, but these are all slight rearrangements along the shared soft modes, which facilitate the adaptation to different substrates. These signature modes are largely conserved across different structures of the same protein as observed in previous studies (Batista et al. 2010, 2011; Krieger et al. 2015; Ponzoni et al. 2018). Secondly, motions in the LTIF regime define the specificity of subfamilies. Members of subfamilies are unified by their shared motions, or mechanisms of actions, in that particular regime, and they are maximally differentiated from other subfamily members precisely by virtue of the differences in their specific motions in this regime.

In summary, robust global dynamics is a unifying feature in favor of the selection of the family fold; whereas LTIF dynamics is the way the specificity requirement copes with common fold. An earlier study demonstrated that global modes are robust to perturbations, which could explain their conservation (Echave and Fernandez 2010). Their robustness to perturbation does not preclude the fact that these modes are also functionally significant, as confirmed in numerous studies. To the extent that functionality is a driving force for selecting structures, these robust modes that are functional would be expected to play a role in the selection or evolution of the structures that favor these modes.

### Convergent versus Divergent Evolution

Despite the wealth of data on well-studied proteins such as TIM barrel proteins, it is still not clear whether their shared fold originates from common ancestry, or results from convergent evolution. Protein folds are presumed to be more susceptible to evolutionary convergence than sequences, but sequence-profile-based phylogenetic analysis can detect evolutionary relationships even among sequentially distant members of a given superfamily, in support of divergent evolution (Theobald and Wuttke 2005). Other studies show that fitness constraints enforce evolutionary paths that preserve protein structure despite sequence divergence down to 30% sequence identity (Gilson et al. 2017). Yet, the currently examined superfamilies contain members with much lower sequence identity, and other studies suggest that there is a limit to amino acid divergence while maintaining the contact topology/fold of the protein (Porto et al. 2005). While the current study cannot ascertain whether the shared structures are maintained during divergent evolution of sequences, or selected by convergent evolution, we clearly distinguish robust signature dynamics shared by family members, as well as LF and LTIF modes that characterize subfamilies. It remains to be established whether the prevalence of robust global motions, and accessibility to selected LTIF modes drive the selection of these folds.

### Future Directions

Current models and methods explain ∼60% of the observed variance in site-specific substitution rates in proteins, highlighting the limitations of state-of-the-art approaches (Echave et al. 2016), which are often based on machine learning methods of sequence analysis and other structure-based considerations such as local packing density and solvent accessibility. Previous analysis demonstrated that local packing density is a major determinant of evolutionary rate, while flexibility, as described by RMSFs is not. ENMs inherently account for packing density, but also provide a higher level of description of the complete topology. Notably, an ENM-based mechanistic study has been shown to account for site-specific evolutionary rates and their relationship with packing density and flexibility (Huang et al. 2014), and another assisted in improving our assessment of the impact of SAVs (Ponzoni and Bahar 2018). While the current study does not aim at inferring causal relationships between structural dynamics and sequence evolution rate, the signature profiles and covariances obtained here upon mathematically exact evaluation and dissection of the coupled dynamics of all residues provide information furthering our understanding of site-specific evolutionary rates or impact of mutations.

Additional studies with *SignDy* by a wide range of users with expertise on particular proteins and families would provide deeper insights into the evolution of dynamics and its importance for function. A reasonable strategy for utilizing *SignDy* in characterizing family/subfamily dynamics vis-à-vis structure and function evolution would be: 1) generate the mode conservation and collectivity profiles for the investigated family (e.g., fig. 5*a* and multiple profiles in supplementary fig. S5, Supplementary Material online); 2) identify the conserved modes (peaks in the same figures, *green curves*) in different regimes; 3) examine the corresponding mode shapes (e.g., figs. 2and 7 and supplementary fig. S6, Supplementary Material online) to 4) identify critical sites responsible for the evolutionarily conserved signature dynamics (minima in global modes) and stability (peaks in HF modes) as well as those susceptible to subfamily specific divergence (in conserved LTIF modes); and 5) generate dendrograms that provide information on dynamics similarities in different regimes, complementing sequence and structure similarities, among family members (fig. 6 and supplementary fig. S7, Supplementary Material online). While subfamily–subfamily spectral distances have been analyzed here based on different frequency windows of structural dynamics (fig. 4 and supplementary fig. S4, Supplementary Material online), computations may be performed for narrower windows or even individual modes, to identify the most discriminative modes and infer new design/engineering principles for alterations of function.

## Materials and Methods

### *SignDy* Architecture and Workflow

*SignDy* is designed as a pipeline composed of seven steps as depicted in figure 1. We present the steps below. Technical details are presented in the supplementary methods, Supplementary Material online, and online tutorials.

1. *Selection of protein family members.* The input to *SignDy* can be entered or generated in three ways: 1) entering a Pfam (Finn et al. 2016) or CATH-Gene3D (Dawson et al. 2017) ID representative of a family; 2) providing a query PDB (Burley et al. 2017) or UniProt (The UniProt 2017) ID, or a sequence in FASTA format, so as to extract the corresponding structural homologues using either existing *ProDy* functions or a new protocol designed to retrieve homologues from the Dali server (Holm and Laakso 2016); or 3) submitting a list of PDB codes for homologous proteins.
2. *Structural alignment and definition of core residues.* This task is conceptually simple but not trivial and critically important. We structurally aligned family members using sequence alignments, the CE structural alignment algorithm (Shindyalov and Bourne 1998), and the alignments output from the Dali server (Holm and Laakso 2016). Comparison of CE and Dali shows the closer superposition of structures achieved by Dali, and hence its use is suggested whenever available (see supplementary methods and supplementary fig. S11, Supplementary Material online). A “core” of *N* residues is identified for each fold, composed of those sites with high-sequence occupancy (>70%), and structurally aligned among all members.
3. *Assessment of sequence and structure similarities among family members and selection of a refined representative set of homologues*. Overrepresented sequences and structures as well as highly dissimilar ones are filtered out as described in the supplementary methods, Supplementary Material online. Typically, the average sequence identity over all pairs within the family is around 0.20 ± 0.12, while pairwise RMSDs remain <7.0 Å. This step yields a refined ensemble of *M* members, including a reference structure *R*. Supplementary figures S1 and S2, Supplementary Material online, display the distributions of the average sequence identities and average RMSDs calculated for the three example families and the 116 CATH superfamilies examined here, respectively.
4. *Evaluation of mode spectra and conserved mechanisms*, using the GNM (Bahar et al. 1997; Li et al. 2016) or ANM (Atilgan et al. 2001; Eyal et al. 2015). Two properties characterize each mode: *shape/mechanism* (i.e., distribution of residue movements), and *frequency/rate*. The modes are ordered from LF (slow/soft, global) to HF (fast, local). We quantify the *mode–mode matches* between *R* and each of the other *M* −1 members of the family/ensemble. The resulting *equivalent modes* for each member are reordered to match the mode order of *R*, and the collectivity of each mode is computed (see supplementary methods, Supplementary Material online).
5. *Identification of signature dynamics*. The spatial mobility of core residues driven by global modes averaged over all members, and its variation across family members, define the “signature dynamics” of the family as illustrated in figure 2*a*–*c* for the LeuT, PBP-1, and TIM barrel families. Another generic property is the cross-correlations or the N×N covariance map between residue motions averaged over all *M* members, which can be evaluated for different frequency windows.
6. *Quantitative assessment of conservation of individual modes and spectral overlap between family members, and between subfamilies.* The level of conservation of mode *k* within a given family is measured by the *mode–mode correlation cosine* computed for the *k*th equivalent mode, averaged over all M(M-1)/2 pairs of members. Another criterion for the extent of similarity between the mode spectra of members AandB,is the spectral overlap, $SO_{\mathrm{ij}}\left(A,B\right),$a cumulative property evaluated for the subset of i≤k≤j modes (see supplementary methods, Supplementary Material online). SO_ij (A,B) is evaluated for different frequency regimes by suitable selection of the indices *i* and *j*. Mode–mode correlation cosines (for individual modes) and spectral overlaps (for sets of modes) both serve as metrics for assessing the conservation of dynamics.
7. *Classification of family members based on their dynamics.* A dynamics-based dendrogram for the family (analogous to a phylogenetic tree) is calculated using the spectral distance between pairs of members *A* and *B*, $d_{\mathrm{ij}}\left(A,B\right)=\;{\text{cos}}^{-1}\left(SO_{\mathrm{ij}}\left(A,B\right)\right),$ as a metric. The differentiation of collective motions in different regimes (i≤k≤j)between the *m* subfamilies is obtained by averaging $d_{\mathrm{ij}}\left(A,B\right)$ values over all members belonging to each pair of subfamilies. These subfamily-subfamily distances conveniently displayed in *m* × *m* matrices for each frequency range i≤k≤j,matrices provide a clear visualization of the conservation or differentiation of different mode regimes across subfamilies (see supplementary methods, Supplementary Material online and fig. 4a-d and supplementary fig. S4a-d, Supplementary Material online). Trees based on structure and sequence distances, use as metrics RMSDs and Hamming distances, $d_{H}\left(A,B\right)=1-\mathrm{seq}\mathrm{identity}\mathrm{fraction}$, respectively. We verified that the RMSDs yield results similar to those obtained with TM score, another measure of structural difference that overcomes some potential problems with the RMSD measure (Zhang and Skolnick 2004) (supplementary fig. S12, Supplementary Material online).

## Data Availability

The data sets used for generating the results are presented in supplementary tables S1–S4, Supplementary Material online.

## Code Availability

The source code for *ProDy* can be found on GitHub at https://github.com/prody/ProDy; last accessed: 04/26/2019. *ProDy* and *SignDy* computing language (*Python*) is essential to extensibility and interoperation with a wealth of modeling tools. Functions for generating ensembles are available in the *ensemble* module; those for generating mode ensembles and analyzing signature dynamics can be found in the *SignDy* module; and those for retrieving data from CATH and Dali are available in the *database* module. All code for generating the results and figures presented in the study are available in the form of tutorials on the *ProDy* website: http://prody.csb.pitt.edu/tutorials/signdy_tutorial/; last accessed: 04/26/2019.

## Supplementary Material

Supplementary data are available at *Molecular Biology and Evolution* online.

## Supplementary Material

msz102_Supplementary_Data

## References

[msz102-B1] Atilgan AR, Durell SR, Jernigan RL, Demirel MC, Keskin O, Bahar I. 2001 Anisotropy of fluctuation dynamics of proteins with an elastic network model. Biophys J. 80 1:505–515. 11159421 10.1016/S0006-3495(01)76033-X PMC1301252

[msz102-B2] Bahar I, Atilgan AR, Demirel MC, Erman B. 1998 Vibrational dynamics of folded proteins: significance of slow and fast motions in relation to function and stability. Phys Rev Lett. 80 12:2733–2736.

[msz102-B3] Bahar I, Atilgan AR, Erman B. 1997 Direct evaluation of thermal fluctuations in proteins using a single-parameter harmonic potential. Fold Des. 2 3:173–181. 9218955 10.1016/S1359-0278(97)00024-2

[msz102-B4] Bahar I, Jernigan RL, Dill KA. 2017. Protein actions: principles and modeling. Garland Sci.

[msz102-B5] Bakan A, Dutta A, Mao W, Liu Y, Chennubhotla C, Lezon TR, Bahar I. 2014 Evol and ProDy for bridging protein sequence evolution and structural dynamics. Bioinformatics 30 18:2681–2683. 24849577 10.1093/bioinformatics/btu336 PMC4155247

[msz102-B6] Bakan A, Meireles LM, Bahar I. 2011 ProDy: protein dynamics inferred from theory and experiments. Bioinformatics 27 11:1575–1577. 21471012 10.1093/bioinformatics/btr168 PMC3102222

[msz102-B7] Batista PR, Pandey G, Pascutti PG, Bisch PM, Perahia D, Robert CH. 2011 Free energy profiles along consensus normal modes provide insight into HIV-1 protease flap opening. J Chem Theory Comput. 7 8:2348–2352. 26606609 10.1021/ct200237u

[msz102-B8] Batista PR, Robert CH, Marechal JD, Hamida-Rebai MB, Pascutti PG, Bisch PM, Perahia D. 2010 Consensus modes, a robust description of protein collective motions from multiple-minima normal mode analysis–application to the HIV-1 protease. Phys Chem Chem Phys. 12 12:2850–2859. 20449375 10.1039/b919148h

[msz102-B9] Bottaro S, Lindorff-Larsen K. 2018 Biophysical experiments and biomolecular simulations: a perfect match? Science 361 6400:355–360. 30049874 10.1126/science.aat4010

[msz102-B10] Burley SK, Berman HM, Kleywegt GJ, Markley JL, Nakamura H, Velankar S. 2017 Protein Data Bank (PDB): the single global macromolecular structure archive. Methods Mol Biol. 1607:627–641. 28573592 10.1007/978-1-4939-7000-1_26 PMC5823500

[msz102-B11] Carnevale V, Raugei S, Micheletti C, Carloni P. 2006 Convergent dynamics in the protease enzymatic superfamily. J Am Chem Soc. 128 30:9766–9772. 16866533 10.1021/ja060896t

[msz102-B12] Cheng MH, Bahar I. 2014 Complete mapping of substrate translocation highlights the role of LeuT N-terminal segment in regulating transport cycle. PLoS Comput Biol. 10 10:e1003879. 25299050 10.1371/journal.pcbi.1003879 PMC4191883

[msz102-B13] Chennubhotla C, Bahar I. 2007 Signal propagation in proteins and relation to equilibrium fluctuations. PLoS Comput Biol. 3 9:1716–1726. 17892319 10.1371/journal.pcbi.0030172 PMC1988854

[msz102-B14] Dawson NL, Lewis TE, Das S, Lees JG, Lee D, Ashford P, Orengo CA, Sillitoe I. 2017 CATH: an expanded resource to predict protein function through structure and sequence. Nucleic Acids Res. 45(D1):D289–D295. 27899584 10.1093/nar/gkw1098 PMC5210570

[msz102-B15] Delarue M. 2008 Dealing with structural variability in molecular replacement and crystallographic refinement through normal-mode analysis. Acta Crystallogr D Biol Crystallogr. 64(Pt 1):40–48. 18094466 10.1107/S0907444907053516 PMC2394787

[msz102-B16] Drew D, Boudker O. 2016 Shared molecular mechanisms of membrane transporters. Annu Rev Biochem. 85:543–572. 27023848 10.1146/annurev-biochem-060815-014520

[msz102-B17] Dror RO, Dirks RM, Grossman JP, Xu H, Shaw DE. 2012 Biomolecular simulation: a computational microscope for molecular biology. Annu Rev Biophys. 41:429–452. 22577825 10.1146/annurev-biophys-042910-155245

[msz102-B18] Dutta A, Krieger J, Lee JY, Garcia-Nafria J, Greger IH, Bahar I. 2015 Cooperative dynamics of intact AMPA and NMDA glutamate receptors: similarities and subfamily-specific differences. Structure 23 9:1692–1704. 26256538 10.1016/j.str.2015.07.002 PMC4558295

[msz102-B19] Echave J, Fernandez FM. 2010 A perturbative view of protein structural variation. Proteins 78 1:173–180. 19731380 10.1002/prot.22553

[msz102-B20] Echave J, Spielman SJ, Wilke CO. 2016 Causes of evolutionary rate variation among protein sites. Nat Rev Genet. 17 2:109–121. 26781812 10.1038/nrg.2015.18 PMC4724262

[msz102-B21] Echave J, Wilke CO. 2017 Biophysical models of protein evolution: understanding the patterns of evolutionary sequence divergence. Annu Rev Biophys. 46:85–103. 28301766 10.1146/annurev-biophys-070816-033819 PMC5800964

[msz102-B22] Eyal E, Lum G, Bahar I. 2015 The anisotropic network model web server at 2015 (ANM 2.0). Bioinformatics 31 9:1487–1489. 25568280 10.1093/bioinformatics/btu847 PMC4410662

[msz102-B23] Felder CB, Graul RC, Lee AY, Merkle HP, Sadee W. 1999 The Venus flytrap of periplasmic binding proteins: an ancient protein module present in multiple drug receptors. AAPS PharmSci. 1 2:E2. 11741199 10.1208/ps010202 PMC2761117

[msz102-B24] Finn RD, Coggill P, Eberhardt RY, Eddy SR, Mistry J, Mitchell AL, Potter SC, Punta M, Qureshi M, Sangrador-Vegas A. 2016 The Pfam protein families database: towards a more sustainable future. Nucleic Acids Res. 44(D1):D279–D285. 26673716 10.1093/nar/gkv1344 PMC4702930

[msz102-B25] Fuglebakk E, Echave J, Reuter N. 2012 Measuring and comparing structural fluctuation patterns in large protein datasets. Bioinformatics 28 19:2431–2440. 22796957 10.1093/bioinformatics/bts445

[msz102-B26] Fuglebakk E, Tiwari SP, Reuter N. 2015 Comparing the intrinsic dynamics of multiple protein structures using elastic network models. Biochim Biophys Acta. 1850 5:911–922. 25267310 10.1016/j.bbagen.2014.09.021

[msz102-B27] Gilson AI, Marshall-Christensen A, Choi JM, Shakhnovich EI. 2017 The role of evolutionary selection in the dynamics of protein structure evolution. Biophys J. 112 7:1350–1365. 28402878 10.1016/j.bpj.2017.02.029 PMC5390048

[msz102-B28] Haliloglu T, Bahar I. 2015 Adaptability of protein structures to enable functional interactions and evolutionary implications. Curr Opin Struct Biol. 35:17–23. 26254902 10.1016/j.sbi.2015.07.007 PMC4688206

[msz102-B29] Hinsen K, Petrescu A-J, Dellerue S, Bellissent-Funel M-C, Kneller GR. 2000 Harmonicity in slow protein dynamics. Chem Phys. 261(1–2):25–37.

[msz102-B30] Hollup SM, Fuglebakk E, Taylor WR, Reuter N. 2011 Exploring the factors determining the dynamics of different protein folds. Protein Sci. 20 1:197–209. 21086444 10.1002/pro.558 PMC3047076

[msz102-B31] Holm L, Laakso LM. 2016 Dali server update. Nucleic Acids Res. 44(W1):W351–W355. 27131377 10.1093/nar/gkw357 PMC4987910

[msz102-B32] Hsieh YC, Poitevin F, Delarue M, Koehl P. 2016 Comparative normal mode analysis of the dynamics of DENV and ZIKV capsids. Front Mol Biosci. 3:85. 28083537 10.3389/fmolb.2016.00085 PMC5187361

[msz102-B33] Huang TT, del Valle Marcos ML, Hwang JK, Echave J. 2014 A mechanistic stress model of protein evolution accounts for site-specific evolutionary rates and their relationship with packing density and flexibility. BMC Evol Biol. 14:78. 24716445 10.1186/1471-2148-14-78 PMC4101840

[msz102-B34] Humphrey W, Dalke A, Schulten K. 1996 VMD: visual molecular dynamics. J Mol Graph. 14 1:33–38. 8744570 10.1016/0263-7855(96)00018-5

[msz102-B35] Juritz E, Palopoli N, Fornasari MS, Fernandez-Alberti S, Parisi G. 2013 Protein conformational diversity modulates sequence divergence. Mol Biol Evol. 30 1:79–87. 22396525 10.1093/molbev/mss080

[msz102-B36] Kazmier K, Claxton DP, McHaourab HS. 2017 Alternating access mechanisms of LeuT-fold transporters: trailblazing towards the promised energy landscapes. Curr Opin Struct Biol. 45:100–108. 28040635 10.1016/j.sbi.2016.12.006 PMC5491374

[msz102-B37] Krieger J, Bahar I, Greger IH. 2015 Structure, dynamics, and allosteric potential of ionotropic glutamate receptor N-terminal domains. Biophys J. 109 6:1136–1148. 26255587 10.1016/j.bpj.2015.06.061 PMC4576161

[msz102-B38] Krishnamurthy H, Gouaux E. 2012 X-ray structures of LeuT in substrate-free outward-open and apo inward-open states. Nature 481 7382:469–474. 22230955 10.1038/nature10737 PMC3306218

[msz102-B39] Li H, Chang YY, Lee JY, Bahar I, Yang LW. 2017 DynOmics: dynamics of structural proteome and beyond. Nucleic Acids Res. 45(W1):W374–W380. 28472330 10.1093/nar/gkx385 PMC5793847

[msz102-B40] Li H, Chang YY, Yang LW, Bahar I. 2016 iGNM 2.0: the Gaussian network model database for biomolecular structural dynamics. Nucleic Acids Res. 44(D1):D415–D422. 26582920 10.1093/nar/gkv1236 PMC4702874

[msz102-B41] Liberles DA, Teichmann SA, Bahar I, Bastolla U, Bloom J, Bornberg-Bauer E, Colwell LJ, de Koning AP, Dokholyan NV, Echave J, et al 2012 The interface of protein structure, protein biophysics, and molecular evolution. Protein Sci. 21 6:769–785. 22528593 10.1002/pro.2071 PMC3403413

[msz102-B42] Liu Y, Bahar I. 2012 Sequence evolution correlates with structural dynamics. Mol Biol Evol. 29 9:2253–2263. 22427707 10.1093/molbev/mss097 PMC3424413

[msz102-B43] Lopez-Blanco JR, Chacon P. 2016 New generation of elastic network models. Curr Opin Struct Biol. 37:46–53. 26716577 10.1016/j.sbi.2015.11.013

[msz102-B44] Luitz M, Bomblies R, Ostermeir K, Zacharias M. 2015 Exploring biomolecular dynamics and interactions using advanced sampling methods. J Phys Condens Matter. 27 32:323101. 26194626 10.1088/0953-8984/27/32/323101

[msz102-B45] Ma J. 2005 Usefulness and limitations of normal mode analysis in modeling dynamics of biomolecular complexes. Structure 13 3:373–380. 15766538 10.1016/j.str.2005.02.002

[msz102-B46] Maguid S, Fernandez-Alberti S, Echave J. 2008 Evolutionary conservation of protein vibrational dynamics. Gene 422(1–2):7–13. 18577430 10.1016/j.gene.2008.06.002

[msz102-B47] Maguid S, Fernandez-Alberti S, Parisi G, Echave J. 2006 Evolutionary conservation of protein backbone flexibility. J Mol Evol. 63 4:448–457. 17021932 10.1007/s00239-005-0209-x

[msz102-B48] Malinauskaite L, Quick M, Reinhard L, Lyons JA, Yano H, Javitch JA, Nissen P. 2014 A mechanism for intracellular release of Na+ by neurotransmitter/sodium symporters. Nat Struct Mol Biol. 21 11:1006–1012. 25282149 10.1038/nsmb.2894 PMC4346222

[msz102-B49] Meireles L, Gur M, Bakan A, Bahar I. 2011 Pre-existing soft modes of motion uniquely defined by native contact topology facilitate ligand binding to proteins. Protein Sci. 20 10:1645–1658. 21826755 10.1002/pro.711 PMC3218357

[msz102-B50] Merkle PS, Gotfryd K, Cuendet MA, Leth-Espensen KZ, Gether U, Loland CJ, Rand KD. 2018 Substrate-modulated unwinding of transmembrane helices in the NSS transporter LeuT. Sci Adv. 4 5:eaar6179. 29756037 10.1126/sciadv.aar6179 PMC5947982

[msz102-B51] Micheletti C. 2013 Comparing proteins by their internal dynamics: exploring structure-function relationships beyond static structural alignments. Phys Life Rev. 10 1:1–26. 23199577 10.1016/j.plrev.2012.10.009

[msz102-B52] Nevin Gerek Z, Kumar S, Banu Ozkan S. 2013 Structural dynamics flexibility informs function and evolution at a proteome scale. Evol Appl. 6 3:423–433. 23745135 10.1111/eva.12052 PMC3673471

[msz102-B53] Perez C, Koshy C, Yildiz O, Ziegler C. 2012 Alternating-access mechanism in conformationally asymmetric trimers of the betaine transporter BetP. Nature 490 7418:126–130. 22940865 10.1038/nature11403

[msz102-B54] Perica T, Kondo Y, Tiwari SP, McLaughlin SH, Kemplen KR, Zhang X, Steward A, Reuter N, Clarke J, Teichmann SA. 2014 Evolution of oligomeric state through allosteric pathways that mimic ligand binding. Science 346 6216:1254346. 25525255 10.1126/science.1254346 PMC4337988

[msz102-B55] Ponzoni L, Bahar I. 2018 Structural dynamics is a determinant of the functional significance of missense variants. Proc Natl Acad Sci U S A. 115 16:4164–4169. 29610305 10.1073/pnas.1715896115 PMC5910821

[msz102-B56] Ponzoni L, Zhang S, Cheng MH, Bahar I. 2018 Shared dynamics of LeuT superfamily members and allosteric differentiation by structural irregularities and multimerization. Philos Trans R Soc Lond B Biol Sci. 373(1749):20170177. 10.1098/rstb.2017.0177 PMC5941172 29735731

[msz102-B57] Porto M, Roman HE, Vendruscolo M, Bastolla U. 2005 Prediction of site-specific amino acid distributions and limits of divergent evolutionary changes in protein sequences. Mol Biol Evol. 22 3:630–638. 15537801 10.1093/molbev/msi048

[msz102-B58] Quiocho FA, Ledvina PS. 1996 Atomic structure and specificity of bacterial periplasmic receptors for active transport and chemotaxis: variation of common themes. Mol Microbiol. 20 1:17–25. 8861200 10.1111/j.1365-2958.1996.tb02484.x

[msz102-B59] Shi Y. 2013 Common folds and transport mechanisms of secondary active transporters. Annu Rev Biophys. 42:51–72. 23654302 10.1146/annurev-biophys-083012-130429

[msz102-B60] Shimamura T, Weyand S, Beckstein O, Rutherford NG, Hadden JM, Sharples D, Sansom MS, Iwata S, Henderson PJ, Cameron AD. 2010 Molecular basis of alternating access membrane transport by the sodium-hydantoin transporter Mhp1. Science 328 5977:470–473. 20413494 10.1126/science.1186303 PMC2885435

[msz102-B61] Shindyalov IN, Bourne PE. 1998 Protein structure alignment by incremental combinatorial extension (CE) of the optimal path. Protein Eng. 11 9:739–747. 9796821 10.1093/protein/11.9.739

[msz102-B62] Skjaerven L, Reuter N, Martinez A. 2011 Dynamics, flexibility and ligand-induced conformational changes in biological macromolecules: a computational approach. Future Med Chem. 3 16:2079–2100. 22098354 10.4155/fmc.11.159

[msz102-B63] Skjaerven L, Yao X-Q, Scarabelli G, Grant BJ. 2014 Integrating protein structural dynamics and evolutionary analysis with Bio3D. BMC Bioinformatics. 15:399. 25491031 10.1186/s12859-014-0399-6 PMC4279791

[msz102-B64] Srivastava A, Nagai T, Srivastava A, Miyashita O, Tama F. 2018 Role of computational methods in going beyond X-ray crystallography to explore protein structure and dynamics. Int J Mol Sci. 19(11):3401. 10.3390/ijms19113401 PMC6274748 30380757

[msz102-B65] Swint-Kruse L, Matthews KS. 2009 Allostery in the LacI/GalR family: variations on a theme. Curr Opin Microbiol. 12 2:129–137. 19269243 10.1016/j.mib.2009.01.009 PMC2688824

[msz102-B66] Tama F, Sanejouand YH. 2001 Conformational change of proteins arising from normal mode calculations. Protein Eng. 14 1:1–6. 11287673 10.1093/protein/14.1.1

[msz102-B67] The UniProt C. 2017 UniProt: the universal protein knowledgebase. Nucleic Acids Res. 45:D158–D169. 27899622 10.1093/nar/gkw1099 PMC5210571

[msz102-B68] Theobald DL, Wuttke DS. 2005 Divergent evolution within protein superfolds inferred from profile-based phylogenetics. J Mol Biol. 354 3:722–737. 16266719 10.1016/j.jmb.2005.08.071 PMC1769326

[msz102-B69] Tirion MM. 1996 Large amplitude elastic motions in proteins from a single-parameter, atomic analysis. Phys Rev Lett. 77 9:1905–1908. 10063201 10.1103/PhysRevLett.77.1905

[msz102-B70] Tirion MM. 2015 On the sensitivity of protein data bank normal mode analysis: an application to GH10 xylanases. Phys Biol. 12 6:066013. 26599799 10.1088/1478-3975/12/6/066013

[msz102-B71] Tiwari SP, Reuter N. 2016 Similarity in shape dictates signature intrinsic dynamics despite no functional conservation in TIM barrel enzymes. PLoS Comput Biol. 12 3:e1004834. 27015412 10.1371/journal.pcbi.1004834 PMC4807811

[msz102-B72] Tiwari SP, Reuter N. 2018 Conservation of intrinsic dynamics in proteins-what have computational models taught us? Curr Opin Struct Biol. 50:75–81. 29287233 10.1016/j.sbi.2017.12.001

[msz102-B73] Tobi D, Bahar I. 2005 Structural changes involved in protein binding correlate with intrinsic motions of proteins in the unbound state. Proc Natl Acad Sci U S A. 102 52:18908–18913. 16354836 10.1073/pnas.0507603102 PMC1323175

[msz102-B74] Tokuriki N, Tawfik DS. 2009 Protein dynamism and evolvability. Science 324 5924:203–207. 19359577 10.1126/science.1169375

[msz102-B75] Townsend PD, Rodgers TL, Pohl E, Wilson MR, McLeish TCB, Cann MJ. 2015 Global low-frequency motions in protein allostery: cAP as a model system. Biophys Rev. 7 2:175–182. 10.1007/s12551-015-0163-9 PMC4432019 26000062

[msz102-B76] Yamashita A, Singh SK, Kawate T, Jin Y, Gouaux E. 2005 Crystal structure of a bacterial homologue of Na+/Cl–dependent neurotransmitter transporters. Nature 437 7056:215–223. 16041361 10.1038/nature03978

[msz102-B77] Yang Z, Lasker K, Schneidman-Duhovny D, Webb B, Huang CC, Pettersen EF, Goddard TD, Meng EC, Sali A, Ferrin TE. 2012 UCSF Chimera, MODELLER, and IMP: an integrated modeling system. J Struct Biol. 179 3:269–278. 21963794 10.1016/j.jsb.2011.09.006 PMC3410985

[msz102-B78] Zhang Y, Skolnick J. 2004 Scoring function for automated assessment of protein structure template quality. Proteins 57 4:702–710. 15476259 10.1002/prot.20264

[msz102-B79] Zheng W, Brooks BR, Thirumalai D. 2009 Allosteric transitions in biological nanomachines are described by robust normal modes of elastic networks. Curr Protein Pept Sci. 10 2:128–132. 19355980 10.2174/138920309787847608 PMC3610319

[msz102-B80] Zou T, Risso VA, Gavira JA, Sanchez-Ruiz JM, Ozkan SB. 2015 Evolution of conformational dynamics determines the conversion of a promiscuous generalist into a specialist enzyme. Mol Biol Evol. 32 1:132–143. 25312912 10.1093/molbev/msu281

