# Supplementary material for: Shared Signature Dynamics Tempered by Local Fluctuations Enables Fold Adaptability and Specificity
Source: Mol Biol Evol. 2019 Apr 27;36(9):2053–68. doi: 10.1093/molbev/msz102 (PMC6736388; doi:10.1093/molbev/msz102)
Supplement: msz102_Supplementary_Data [file msz102_supplementary_data.zip › SignDy-SI.pdf]

Supplementary Information for

Shared signature dynamics tempered by local fluctuations enables fold adaptability and specificity

She Zhang*, Hongchun Li*, James M. Krieger*, and Ivet Bahar†

Department of Computational and Systems Biology, School of Medicine, University of Pittsburgh, 3501 Fifth Ave, Suite 3064 BST3, Pittsburgh, PA 15260, USA

** Equal contribution*

† Corresponding author: E-mail: bahar@pitt.edu

Supplementary Methods

DATASETS

Dataset of CATH Superfamilies

We considered the 175 most populated superfamilies in the CATH database (Dawson, et al. 2017), and selected 116 comprised of a total of 26,899 proteins, referred to as Dataset 4, after eliminating the close structural homologs (RMSD ≤ 1 Å) using single-linkage clustering, as well as outliers (RMSD ≥ 10 Å with respect to the reference; see **Pre-processing of dataset** below for details of these filters), and superfamilies with less than 50 representative members.

We structurally aligned all the members within each superfamily using the CE algorithm (Shindyalov and Bourne 1998), which we implemented in *ProDy*. **Supplementary table S4** lists the superfamilies in Dataset 4 and their properties. For each superfamily, we calculated the pairwise sequence identities and RMSDs between all pairs of members and evaluated the average values and standard deviations. The histograms in **supplementary figure S2a-b** show the sequence and structure similarities among the members of each superfamily.

The average of pairwise sequence identities within the superfamilies are around 0.20 meaning that the sequences within superfamilies are quite divergent; whereas the average RMSDs are ~ 4.0 Å, indicating strong structural homology within superfamilies (especially since structures with RMSD < 1.0 Å have been filtered out). Yet, we found a correlation between sequence identity and RMSD (*r* = -0.68; **supplementary fig. S2c**). <RMSD> showed no dependency on the size of proteins, size being defined as the number of structurally aligned residues (**supplementary fig. S2d**), as expected from the normalization of this quantity with respect to the number of residues, by definition.

Datasets for LeuT, PBP and TIM barrel fold families

LeuT. Despite their common fold, many members of the LeuT fold family share low pairwise sequence similarities (< 20% identity). We have manually collected 92 PDB structures composed of 104 protomers with the LeuT fold. The initial ensemble consists of 50 LeuTs and their mutants, 14 DATs, 2 MhsTs, 6 Mhp1s, 1 vSGLT, 6 BetPs, 4 CaiTs, and 7 AdiCs. 85 protomers were selected after removing the LeuT structures that were almost identical (≤ 0.3 Å RMSD) to the reference (PDB ID: 2A65) (Yamashita, et al. 2005). See **supplementary table S1** for more details, and **supplementary figure S1a** for the respective distributions of pairwise sequence identities, structural RMSDs and biological function among the members of the LeuT family.

PBP-1. Individual PBP-1 domain structures were selected and aligned with Dali using a structure of the N-terminal domain (NTD) of AMPA-type ionotropic glutamate receptor (iGluR) paralogue GluA2 (chain A from PDB ID: 3H5V) (Jin, et al. 2009) as the query. This search yielded a set of 2,291 chains from 977 structures including isolated domains and whole receptors. We filtered out those results with Dali Z score

below 10 and less than 50% coverage, resulting in 971 chains from 451 structures. We further refined the set by applying an RMSD filter that removed redundant structures within 1.0 Å RMSD from others in the ensemble, as well as the outliers (≥ 10.0 Å RMSD from all others). This led to an ensemble with 379 members (**supplementary table S2**), including iGluR NTDs, class C G-protein coupled receptor (GPCR) and natriuretic peptide receptor ligand-binding domains, and bacterial periplasmic binding proteins (PBPs) and transcription regulators (TRs). **Supplementary figure S1b** displays the histograms of pairwise sequence identities, structural RMSDs and biological function for PBP family members.

TIM barrels. TIM barrel structures were selected and aligned by Dali using the triose phosphate isomerase (TIM) structure with PDB ID 8TIM (chain B) (Banner, et al. 1975) as the query. The search yielded a total of 1,070 structures. Among them, 455 were filtered out by requiring the following criteria to be satisfied: RMSD > 1 Å with respect to the query structure; Dali Z score > 10; and coverage > 0.7. Among the remaining 615 structures, 14 could not be aligned using the mapping information from Dali, which led to 601 structures. As an additional filter, we excluded members from all pairs outside the range 1 < RMSD < 10 Å. This led to an ensemble of 290 conformations and the multiple sequence alignment (MSA) columns were trimmed to ensure column occupancies of 0.7 or higher, resulting in 180 columns corresponding to core residues (see **supplementary table S3**). **Supplementary figure S1c** displays the distribution of sequence identities, structural RMSDs and biological function among the members of the TIM barrel family.

DETAILED *SIGNDY* WORKFLOW

The *SignDy* workflow is composed of 7 steps as outlined in the main text and illustrated in **figure 1**. We present below more details on each step, and in italics, we provide *ProDy* classes and functions for different operations. The code and documentation can be obtained via our website *prody.csb.pitt.edu*.

Automated retrieval of structural data for queried families (step 1 in fig. 1). The dataset can be retrieved by two major means depending on the type of queried families: sequence or structure homologues. For sequence homologues, users can use the *ProDy* function *blastPDB* to extract structures from the PDB for a given (reference) sequence, or enter a Pfam ID to retrieve the corresponding MSA (using *fetchPfamMSA*) and the associated PDB structures (using *fetchPfamPDBs*). For structural homologues, which may be sequentially distant, a structure-based pipeline using the Dali server (Holm and Laakso 2016) has been developed. This newly implemented function *searchDali* selects and aligns proteins with similar structures. We also have a *CATHDB* class that allows users to explore the CATH database (Dawson, et al. 2017) and select PDB IDs from particular Classes, Architectures, Topologies and Homologous superfamilies. Finally, users have the option of submitting a user-selected set of PDB structures and aligning them later. One member is selected as reference structure. This may be either user-defined, or selected based on pre-defined criteria, such as minimal RMSD from all others after optimal structural alignment.

Pre-processing of the dataset to generate a structurally aligned ensemble (steps 2-3 in fig. 1). Structural alignment is achieved using (i) the MSA obtained from Pfam or BLAST, or generated using the *ProDy* function *alignSequencesByChain,* which provides a wrapper to ClustalW programs (Larkin, et al. 2007), or pairwise sequence alignment based on methods from Biopython (Cock, et al. 2009); (ii) the alignment

from the Dali server (Holm and Laakso 2016); or (iii) the CE structural alignment algorithm (Shindyalov and Bourne 1998) integrated in *ProDy* via the functions *ccealign* and *getCEAlignMapping*. Our recommendation is to use DALI-aligned sets, if available for the query family (which we did for TIM and PBP families), as these structural alignments have been refined by elaborate methods. In case DALI alignments are not available (as in the case of the manually curated set of LeuT fold family transporters, or the CATH superfamilies analysed here), *SignDy* offers the fully automated and integrated CEAlign tool.

PDB IDs and atom-atom alignment data are fed to *buildPDBEnsemble*, which produces the ensemble of 3D coordinates for the superposed structures and has options for choosing the alignment method. The ensemble can be refined by filtering out outliers and overrepresented members (rows in the MSA) based on pairwise sequence identities (evaluated with *buildSeqidMatrix*). Likewise, underrepresented residues (below a column-occupancy threshold of 0.7 in MSA) are removed from the core structure by *trimPDBEnsemble*, but are still accessible for use as environment in the calculation of mode spectra and in the comparison of family members (see below). Structural outliers, each with a large RMSD with respect to the reference, are discarded. Overrepresented structures are identified by evaluating pairwise RMSDs with the ensemble's method *getRMSD*, and single-linkage hierarchical clustering is used to separate groups, within each of which only the most complete structure is retained (all these filters are implemented in *refineEnsemble*). The lower and upper threshold RMSDs used as default for the respective criteria are 1 and 10 Å. If no reference structure was selected, then the first structure in the list provided to *buildPDBEnsemble* will be treated as reference by default.

Calculation of mode spectra and sorting of modes (step 4 in fig. 1). GNM or ANM analyses are performed for each member, using its complete structure composed of the core (shared by all members) and other residues (specific to members) using the system-environment framework implemented in the *reduceModel* function. The effect of the environment on the core dynamics is modelled therein by adopting a modified Connectivity (GNM) or Hessian (ANM) matrix for the core (Hinsen, et al. 2000; Ming and Wall 2005; Zheng and Brooks 2005; Dutta, et al. 2015). In this way, we identify the mode spectrum for each family member. The high-throughput examination of protein family dynamics is possible because of the efficiency of ENMs. ENM calculations are automatically performed for all members of the ensemble using the *calcEnsembleENMs* function. Previous comparisons with molecular dynamics simulations have consistently demonstrated that ENM yields a comparable, if not better, description of a protein's collective dynamics and covariance (Ahmed, et al. 2010; Romo and Grossfield 2011; Leioatts, et al. 2012; Gur, et al. 2013).

Identification of the mode spectrum using the GNM means the determination of the complete orthonormal set of N-1 modes' shapes and frequencies for a network model of N nodes, with each node representing a residue. Mode shapes and frequencies are described by the respective eigenvectors ($\boldsymbol{v}_k$, 1 ≤ k ≤ N-1) and eigenvalues (λ_k), ordered in ascending order, e.g. $\lambda_1 \leq \lambda_2 \leq \lambda_3 \leq \cdots \leq \lambda_{N-1}$) of the N × N connectivity matrix ($\boldsymbol{\Gamma}$) that describes the network topology. The spatial correlations between the movements of the N residues are described by the covariance matrix $\mathbf{C}_{GNM}$. $\mathbf{C}_{GNM}$ scales with the inverse of $\boldsymbol{\Gamma}$, such that

$$\mathbf{C}_{GNM} = \sum_{k=1}^{N} \left(\frac{1}{\lambda_k}\right) \boldsymbol{v}_k \boldsymbol{v}_k^{\mathrm{T}} \quad (1)$$

where $\boldsymbol{v}_k^{\mathrm{T}}$ is the transpose of $\boldsymbol{v}_k$. The inverse eigenvalue $1/\lambda_k$ provides a measure of the weight (or amplitude) of each mode. The cumulative weight for a subset of *n* modes is $\sum_{k=1}^{n} 1/\lambda_k$. In the ANM analysis, these are replaced by 3*N*-6 eigenvectors and eigenvectors of the Hessian $\mathbf{H}$ and the *3N* × *3N* covariance matrix $\mathbf{C}_{ANM}$ (Bahar, et al. 2017). The mean square fluctuations (MSF) are given by the diagonal elements of the covariance matrix and their square roots are called root-mean-square fluctuations (RMSF).

Because of the structural variations, the order (or relative frequencies) of the modes may vary among family members. Pairwise comparisons of the mode spectra of family members necessitate the identification of the equivalent modes. We accomplish *mode-mode matching* as a linear assignment problem (Kuhn 1955). Accordingly, we first calculate the correlation cosine, $\rho_{kl}(A,B) = \boldsymbol{v}_{\boldsymbol{k}}^{A} \cdot \boldsymbol{v}_{\boldsymbol{l}}^{B}$, between each pair of modes *k* and *l* belonging to proteins *A* and *B*, then evaluate the cost of matching them as [1 - $\rho_{kl}(A,B)$], and finally select the set of pairs that minimizes the total cost.

Evaluation of signature dynamics (step 5 in fig. 1). The signature dynamics is defined by global modes of motions (e.g. $\langle \boldsymbol{v}_k \rangle$, *1 ≤ k ≤ 3*) shared by family members, and/or the $\langle MSF \rangle$ profile of residues driven by a selected subset of global modes or all modes, and the cross-correlations $\langle \mathbf{C}_{ij} \rangle$ between residue fluctuations given by the off-diagonal elements of $\mathbf{C}_{GNM}$ (or the trace of the *ij*th off-diagonal 3 × 3 super-element of $\mathbf{C}_{ANM}$). The angular brackets designate the averages over all members of the family. These properties describe the *generic* behaviour of the family. Note that each mode describes a fully symmetric fluctuation; so $\boldsymbol{v}_k$ and $-\boldsymbol{v}_k$ represent the same eigenvector; and eigenvectors are assigned the same sign as their counterparts in the reference structure before evaluating the averages $\langle \boldsymbol{v}_k \rangle$ over family members.

The departures of the individual members from the generic behaviour are given by the standard deviations $\Delta\boldsymbol{v}_k$, ΔMSF and $\Delta\mathbf{C}_{ij}$, displayed by a band around the mean values ($\Delta\boldsymbol{v}_k$ and ΔMSF) or by an additional *N* × *N* map ($\Delta\mathbf{C}_{ij}$). **Figure 2a-c** illustrates the signature profiles for the three cases studied.

We also evaluated the collectivity of the modes and examined to what extent the conservation or differentiation of modes among family or subfamily members relate to their collectivity. The collectivity of mode *k* was evaluated using the following definition (Brüschweiler 1995):

$$\kappa_k = \frac{1}{N} \exp\left\{ -\sum_{n=1}^{N} v_{k,n}^2 \ln v_{k,n}^2 \right\} \tag{2}$$

where *N* is the number of residues, and $u_{k,n}^2$ designates the square displacement of residue *n* along mode *k*, normalised such that $\sum_{n=1}^{N} v_{k,n}^2 = 1$.

Spectral overlap and mode-mode correlations as metrics for similarity between the intrinsic dynamics of family members (step 6 in fig. 1). An important consideration has been the definition of metrics for quantitative comparisons of the fluctuation patterns of proteins. A previous study of a total set of 189

domains, representing four main SCOP (Murzin, et al. 1995) classes has shown that the root-mean-square fluctuation (RMSF) of residues is not a good metric for probing the (dis)similarities in protein dynamics (Fuglebakk, et al. 2012). Instead several alternative metrics have been tested; the importance of examining the directions of fluctuations and their covariance has been emphasized. In the present study, as a measure of the degree of similarity between the global mode spectra of structures *A* and *B*, we use the spectral overlap (Hess 2002). The spectral overlap provides a robust and easy-to-compute metric, as a function of the entire set of eigenvalues and eigenvectors that underlie the mode spectrum, and can be evaluated for subsets of modes. The cumulative spectral overlap based on *k* low-frequency modes (i.e. mode index in the range [1, *k*]) predicted by the GNM is defined as

$$SO_{1k}(A,B) = 1 - \left[\frac{\sum_{i=1}^{k}(\sigma_i^A + \sigma_i^B) - 2\sum_{i=1}^{k}\sum_{l=1}^{k}(\sigma_i^A\,\sigma_l^B)^{\frac{1}{2}}(\boldsymbol{v}_i^A \cdot \boldsymbol{v}_l^B)^2}{\sum_{i=1}^{k}(\sigma_i^A + \sigma_i^B)}\right]^{\frac{1}{2}} \quad (3)$$

where the subscripts in $SO_{1k}(A,B)$ indicate the frequency range (from mode 1 to mode *k*), σ_i^A designates the ith eigenvalue of $\mathbf{C}_{GNM}$ for protein *A*, and $\boldsymbol{v}_i^A$ is the corresponding eigenvector. Note that $\lambda_i^A = 1/\sigma_i^A \cdot SO_{1k}(A,B)$ varies in the range [0, 1]. The upper limit corresponds to the full spectrum of $k = N - 1$ modes which forms a complete basis set for all accessible motions in the *N*-dimensional conformational space. For each superfamily and a given *k*, the spectral overlap is averaged over all $M(M-1)/2$ pairs of *A* and *B*.

A detailed analysis of the extent of differentiation between the individual modes of family members is performed by evaluating the frequency dispersion of their global modes, and the *correlation cosines,* also called *mode-mode overlaps,* averaged over all $M(M-1)/2$ pairs

$$\langle cc_k \rangle = \frac{2}{M(M-1)}\sum_{A}\sum_{B\neq A}\boldsymbol{v}_k^A \cdot \boldsymbol{v}_k^B \quad (4)$$

Note that the mode number *k* refers to the rank-ordered index determined after identifying the optimal matches between the mode spectra of family members, as described in step 4.

Construction of dynamics-based dendrograms (step 7 in fig. 1). The spectral distance, $d_{1k}(A,B)$, between the *k* global modes of *A* and *B* is defined by the arc cosine $d_{1k}(A,B) = \cos^{-1}(SO_{1k}(A,B))$ and that among all members of a given family is evaluated as

$$\langle d_{1k} \rangle = \frac{2}{M\,(M-1)}\sum_{A}\sum_{B\neq A}\cos^{-1}(SO_{1k}(A,B)) \quad (5)$$

The *M* × *M* distance matrix d_{1k}(*A, B*) with *k* = 5 is used as metric for classifying family members based on their global mode spectra.

Alternatively, one can generate dynamics-based dendrograms for different frequency regimes, e.g. modes $i < k \leq j$ (see for example the results for PBP based on modes in the LTIF regime in **supplementary figure S7c**)

The dendrograms (**fig. 6 and supplementary fig. S7**) are constructed using the neighbour joining (NJ) (Saitou and Nei 1987) or Unweighted Pair Group Method with Arithmetic Mean (UPGMA) (Sokal 1958) method. Similar trees based on sequence and structure dissimilarities allow for comparing the differentiations of sequence, structure and dynamics among the members of the family. Here we adopted the RMSDs after structural alignment as structure distance, and the Hamming distance $d_H(A, B)$ (normalized by the number of columns in the MSA) as sequence distance between members *A* and *B*. The trees were saved in Newick (.nwk) format and visualised using the Interactive tree of life (iTOL) server (Letunic and Bork 2016). Functional families are coloured using custom Python scripts.

Evaluation of the conservation/differentiation of modes in different frequency regimes and relationship to functional classification of subfamilies. Toward identifying which particular modes, or modes in which frequency regime, unify members *within* subfamilies, while ensuring maximal differentiation *between* subfamilies themselves, we have constructed *m* × *m* matrices, the entries of which provide a quantitative measure of the extent of similarity, or differences, in the dynamics of subfamilies. We used as metric the distances deduced from the generalization of equation 5

$$\langle d_{ij} \rangle_{m_p, m_s} = \frac{1}{m_p \, m_s} \sum_{A=1}^{m_p} \sum_{B=1}^{m_s} \cos^{-1}(SO_{ij}(A, B)) \qquad (6)$$

Here $\langle d_{ij} \rangle_{m_p, m_s}$ designates the distance between subfamilies *p* and *s*, composed of m_p and m_s members respectively, based on the similarity of their modes $i < k \leq j$. These elements form the off-diagonal elements of the subfamily-subfamily distance matrices. The diagonal elements on the other hand, where the double summation is performed over all elements in the same family (*p* = *s*), are calculated using

$$\langle d_{ij} \rangle_{m_p, m_p} = \frac{1}{m_p \left(m_p - 1 \right)} \sum_{A=1}^{m_p} \sum_{B=1, B \neq A}^{m_p} \cos^{-1}(SO_{ij}(A, B)) \qquad (7)$$

Figure 4 and **supplementary figure S4** illustrate the respective results for TIM and PBP-1 families. The functions of TIM barrel fold subfamilies were identified by mapping against CATH superfamilies. For the PBP-1 case, we used a clustering approach based on a sequence tree to identify subgroups with the function *findSubgroups* and used the names of representative members to assign functional subfamilies.

Supplementary Figures

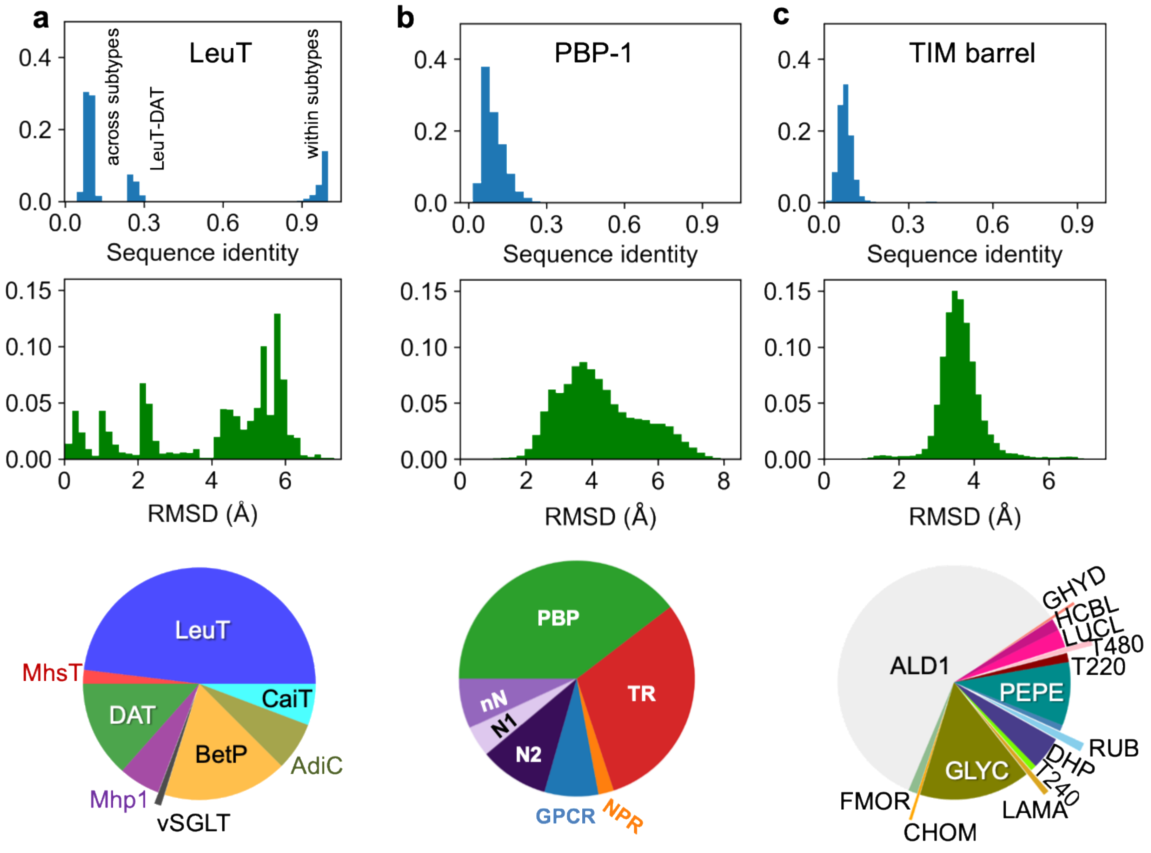

Supplementary Fig. S1. Sequence, structure and function properties of LeuT, PBP-1 and TIM barrel fold family members included in Datasets 1-3. Distributions of average fractional sequence identities and average structural RMSDs within the LeuT (**a**), PBP-1 (**b**), and TIM-barrel (**c**) fold families (*upper and middle panels*), and biological functions of family members (*pie charts, lower panels*). The sequence identity histogram for the LeuT family of transporters (**a**) shows three groups, at 0.10, 0.28 and 0.95, which correspond to the low similarity between distinct subtypes, the intermediate similarity between LeuT/DAT pairs, and the near identity of transporters of the same subtype. PBP-I and TIM barrel family members (**b-c**) have highly dissimilar sequences (with <sequence identity> of 0.11 and 0.085, respectively), while their <RMSD> values are 4.25 and 3.64 Å. *Abbreviations*: GLYC, glycosidases; AdiC, arginine/agmatine antiporter; ALD1, Aldolase class I; BetP, glycine betaine transporter; CaiT, carnitine/butyrobetaine antiporter; CHOM, copper homeostasis (CutC) domain; DAT, dopamine transporter; DHP, dihydropteroate synthase-like; FMOR, FMN-linked oxido-reductase; GHYD, glycoside hydrolase, family 3, N-terminal domain; GPCR, G-protein coupled receptor; HCBL, homocysteine-binding-like domain; LAMA, D-lysine 5,6-aminomutase a-subunit; LeuT, leucine transporter; LUCL, luciferase-like domain; Mhp1, benzyl-hydantoin transporter; MhsT, multi-hydrophobic amino acid transporter; MHYD, metal-dependent hydrolases; MTMB, monomethylamine methyltransferase MtmB; nN, non-NMDA iGluR; N1, GluN1 NMDA iGluR subunit; N2, GluN2 NMDA iGluR subunit; PBP, periplasmic binding protein; PEPE, phosphoenolpyruvate-binding domain; RUB, ribulose bisphosphate carboxylase large subunit C-terminal domain; T220, TIM barrel superfamily 3.20.20.220; T240, TIM barrel superfamily 3.20.20.240; T480, TIM superfamily 3.20.20.480; T540, TIM barrel superfamily 3.20.20.540; TR, transcription regulator; vSGLT, *Vibrio* sodium/galactose transporter.

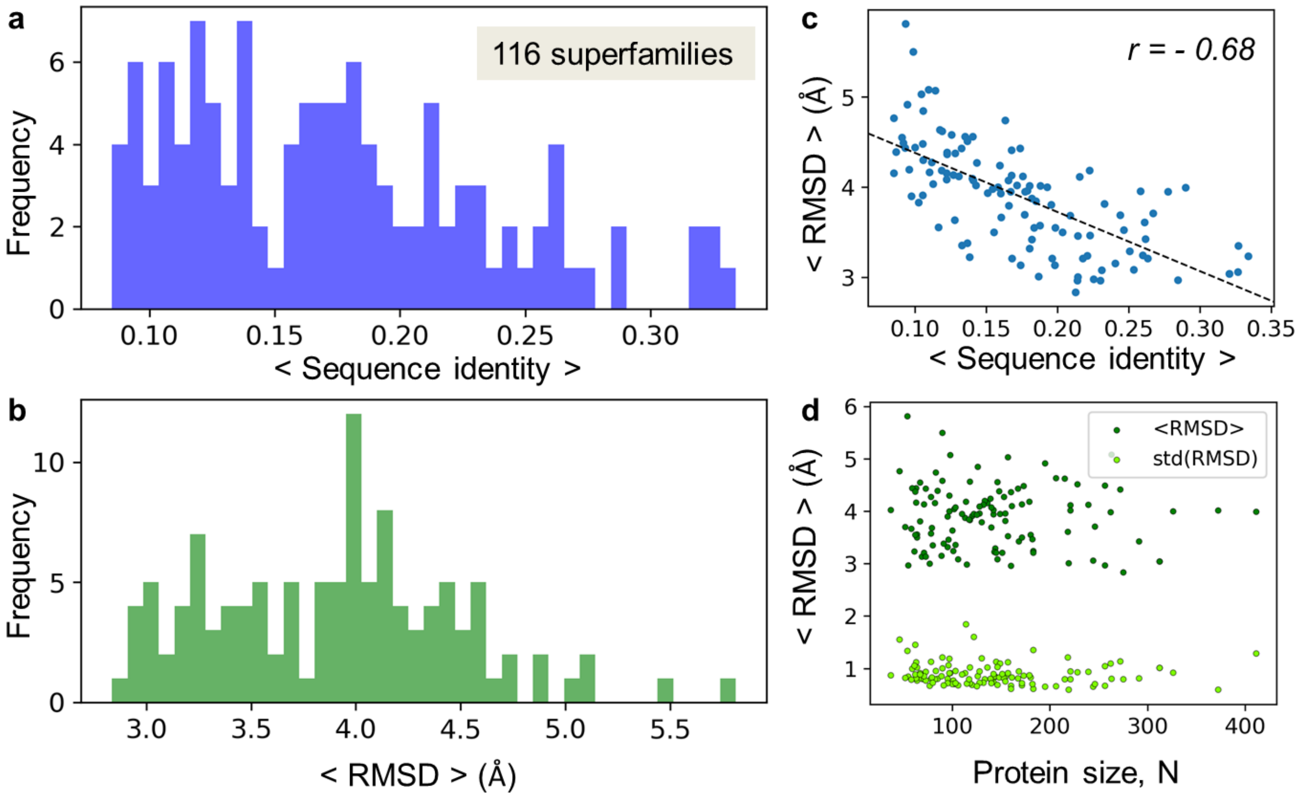

Supplementary Fig. S2. Sequence and structure similarities between superfamily members, evaluated for 116 CATH superfamilies. Panels **a** and **b** display histograms (number distributions) of the average pairwise sequence identities (**a**) and pairwise RMSDs (**b**), computed for the membership of each superfamily. For each superfamily, the pairwise sequence identities and pairwise RMSDs after optimal alignment of pairs of structures were evaluated. The quantities along the abscissa represent the averages, <sequence identity> and <RMSD>, over all pairs in a given superfamily, and the distributions are shown for all superfamilies. The sequence identities averaged over all pairs of members vary in the range 0.08 - 0.35 with a mean value of 0.17 ± 0.06, while the corresponding pairwise RMSDs vary in the range 2.80 - 5.84 Å, with a mean value of 3.90 ± 0.59 Å, indicating low sequence identity despite structural similarity. (**c**) <RMSD> decreases with increasing <sequence identity>. (**d**) The family-averaged RMSDs and their standard deviations are insensitive to the size of the core structures.

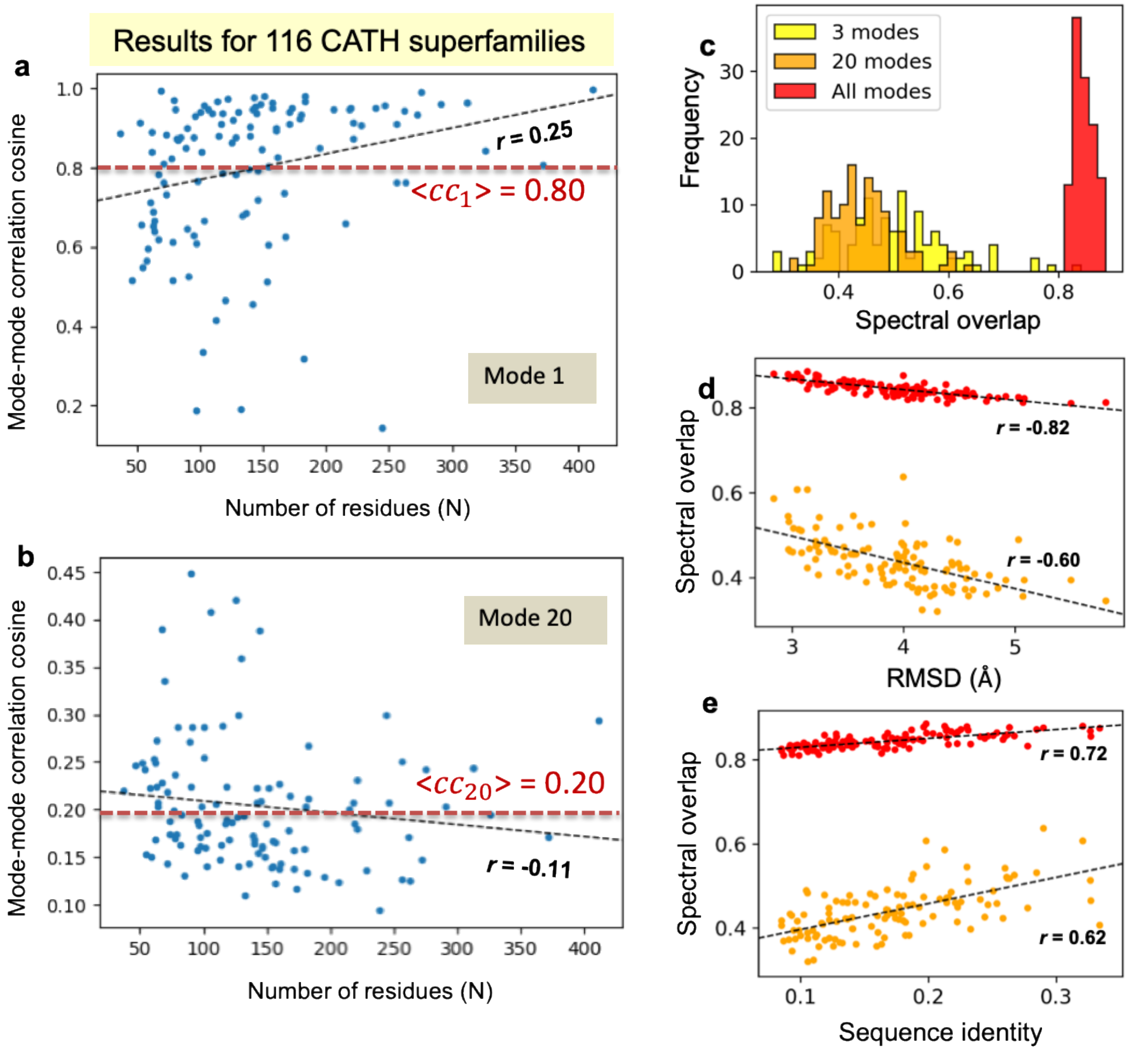

Supplementary Fig. S3. Decrease in mode-mode correlation among family members with increasing mode number, and relationships between the spectral overlap of modes, the structural RMSD between family members and their sequence distance. (**a**-**b**) Mode-mode correlation cosines, $cc_k(f) = \langle cc_k(A, B)\rangle$ averaged over all pairs of superfamily members A and B, plotted as a function of the size of the proteins (or number *N* of structurally aligned residues) in a given CATH superfamily. Results are shown for mode $k = 1$ (**a**), and $k = 20$ (**b**). Each dot represents the result for a given CATH superfamily *f*. A weak dependence on the number of residues *N* is observed, confirming the insensitivity of the observed behaviour to protein size. Comparison of the two panels (ordinates) shows a significant difference in the level of conservation of modes 1 and 20. The *red dashed* curve in each panel shows the average of $cc_k(f)$ over all families: 0.80 ± 0.19 and 0.20 ± 0.07 for the two respective modes. **(c-e) Dependency of cumulative spectral overlap on frequency regime, sequence similarities and structure similarities among superfamily members.** (**c**) Distribution of spectral overlaps between superfamily members based on global ($k \leq 3$), low frequency ($k \leq 20$), and all ($k \leq N - 1$) modes. (**d**-**e**) Dependency of spectral overlap on structural similarity (RMSD) (**d**) and sequence identity (**c**) among members, shown for low frequency (*orange dots*) and all (*red dots*) modes. Inclusion of all modes yields higher overlaps and more pronounced dependencies. The broad dispersion of data at low frequency modes indicate their weaker dependency on the sequence and structural variations.

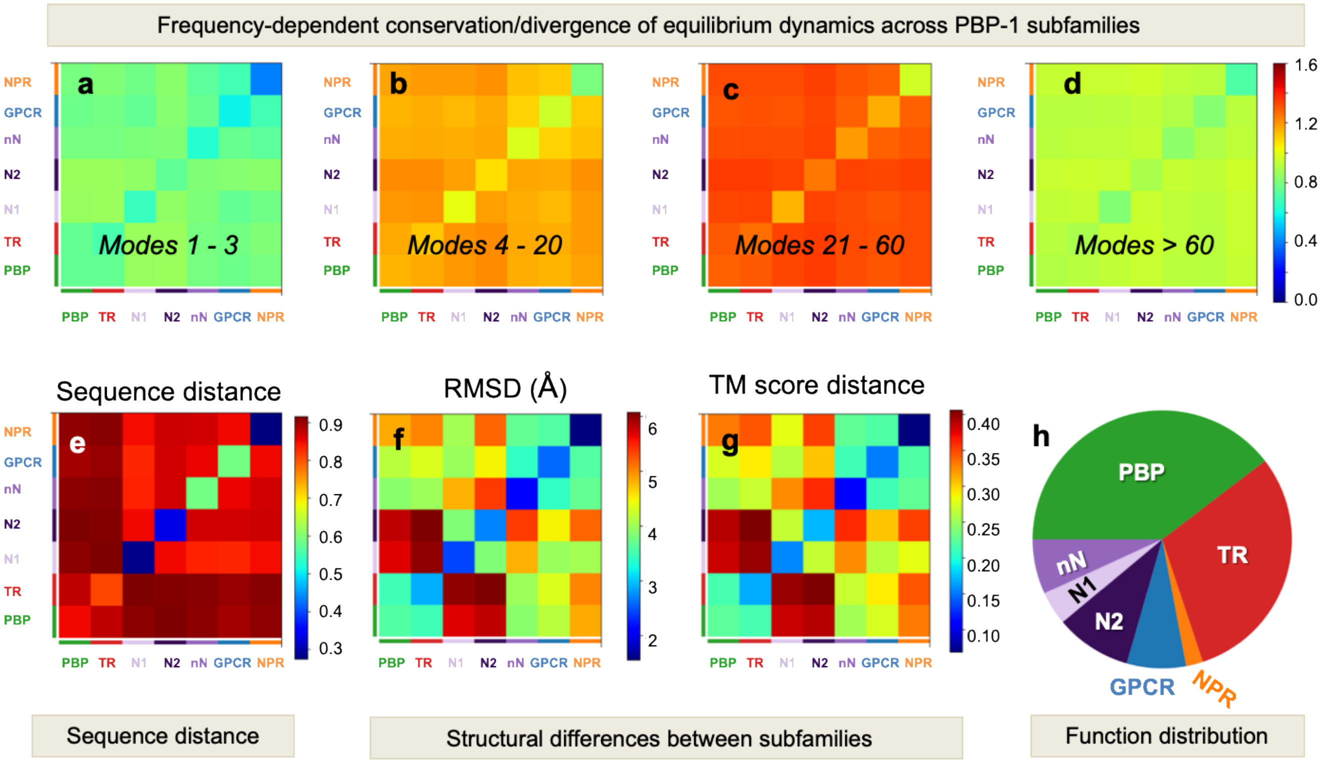

Supplementary Fig. S4. LITF modes maximally discriminate the subfamilies of PBP-1 structures. (**a**-**d**) Mean spectral distances between and within six PBP-1 subfamilies (see full names in **supplementary figure S1** and **supplementary table S2**) are shown for four groups of GNM modes characterized by four frequency regimes: global (modes 1-3; **a**), LF (modes 4-20; **b**), LTIF (modes 21-60; **c**) and HF (modes >60; **d**). The diagonal terms indicate mean values of spectral distances among members within each of the subfamilies. The off-diagonal terms indicate mean values between pairs of subfamilies. The matrices are coloured by the range of spectral distances with low and high values of mean spectral distances coloured *blue* and *red* respectively. The labels of the subfamilies are coloured the same as **supplementary table S2**. (**e**-**h**) Distances between the sequences and structures of these groups are shown for comparison. The sequence distance (**e**) shows the clearest separation of subfamilies and was used to divide the family members into the subfamilies shown in **h** (reproduced from **supplementary figure S1b**). The structural distance is measured in two ways: RMSD (**f**) and TM score (**g**), which give similar results.

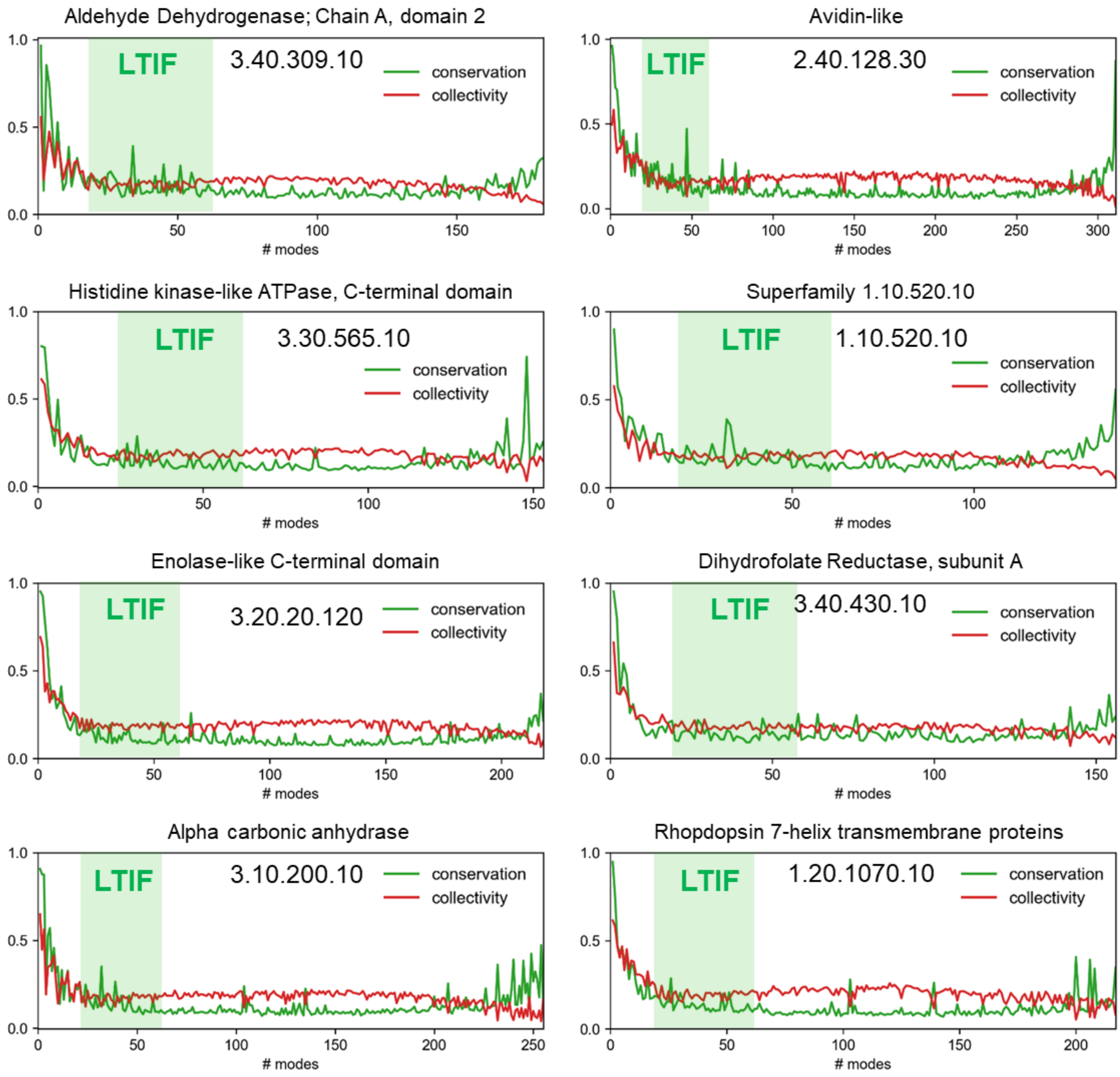

Supplementary Fig. S5. Mode conservation of GNM modes correlate with collectivity in different mode frequency regimes. Mean value of mode conservation (*green*) and collectivity (*red*) for eight selected CATH superfamilies in **supplementary table S4** are shown as a function of mode numbers respectively. The names of CATH domains are shown on the top of panels and the corresponding CATH IDs are shown below the names. The mode conservation decreases as the collectivity decreases at the global and low frequency regimes. While, the correlation reverses at the frequency regimes of LTIF and fast modes. The *green mask* indicates the potential range of the LTIF regime.

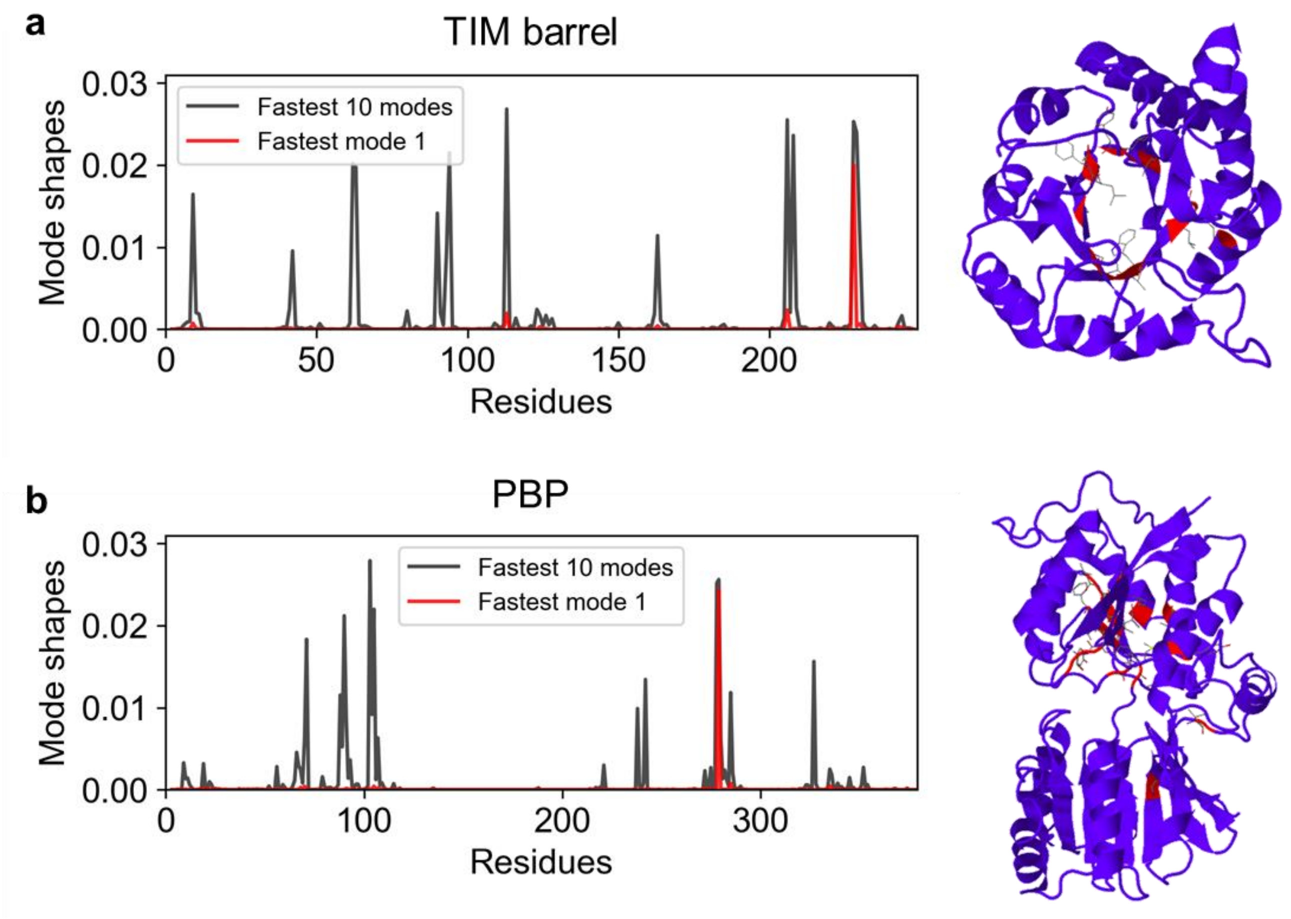

Supplementary Fig. S6. High peaks of the fastest 10 modes of TIM barrel and PBP-1 structures are located in the core of the structures. The mean-squared-fluctuations of the fastest 10 modes and the fastest mode are shown in *grey* and *red* curves for (**a**) TIM barrel (PDB code: 8TIM, chain B) and (**b**) PBP-1 (PDB code: 3H5V, chain A) structures respectively. Ribbon diagrams of the structures are coloured *red* for those residues having a high peak in the fastest 10 modes while other residues are coloured *blue*.

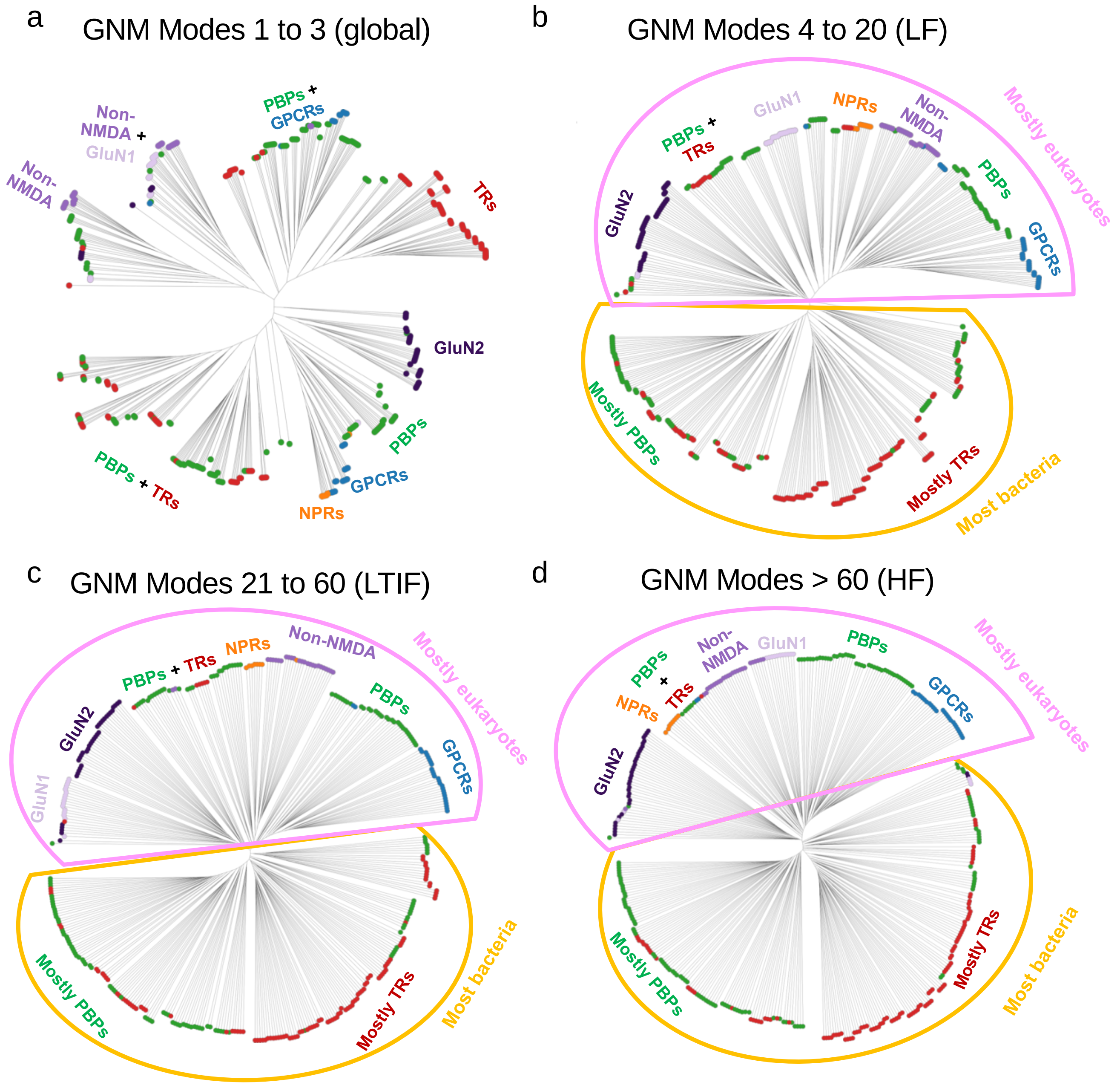

Supplementary Fig. S7. Categorization of family members based on their dynamics in different frequency regimes. Dendrograms for PBP-1 fold family members are shown for four groups of GNM modes characterized by four frequency regimes: global (modes 1-3; **a**), slow (modes 4-20; **b**), LTIF (modes 21-60; **c**) and fast (modes >60; **d**). Subfamilies are coloured as in **figure 6** and **supplementary table S2**. The regions encircled in pink and gold highlight the ability to mostly differentiate bacteria from eukaryotes in panels **b** to **d** as in the sequence tree in **figure 6a.**

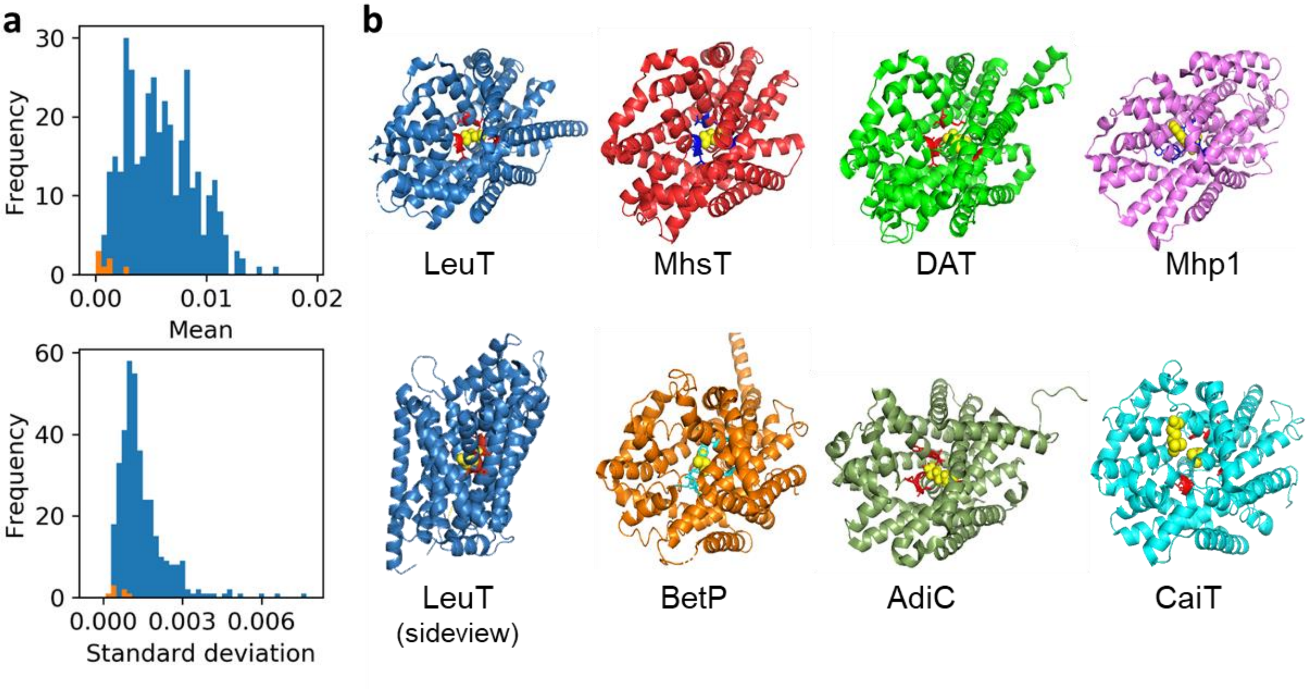

Supplementary Fig. S8. The substrate-binding pocket of LeuT-fold transporters shows minimal fluctuations. (**a**) Distribution of the mean values (*top*) and standard deviations (*bottom*) for square fluctuations of all residues (*blue bars*) and only substrate-binding site residues (*orange bars*). (**b**) Structures of most transporter subtypes are shown from the extracellular side to illustrate the binding pocket. LeuT is shown from the side. vSGLT is not shown because the only available PDB structure in that case is a substrate-free transporter. The corresponding PDB codes are: 2A65 (Yamashita, et al. 2005) for LeuT, 4US4 (Malinauskaite, et al. 2014) for MhsT, 4XP9 (Wang, et al. 2015) for DAT, 4D1D (Simmons, et al. 2014) for Mhp1, 2XQ2 (Watanabe, et al. 2010) for vSGLT, 4LLH (Perez, et al. 2014) for BetP, 5J4I (Ilgü, et al. 2016) for AdiC, and 4M8J (Kalayil, et al. 2013) for CaiT.

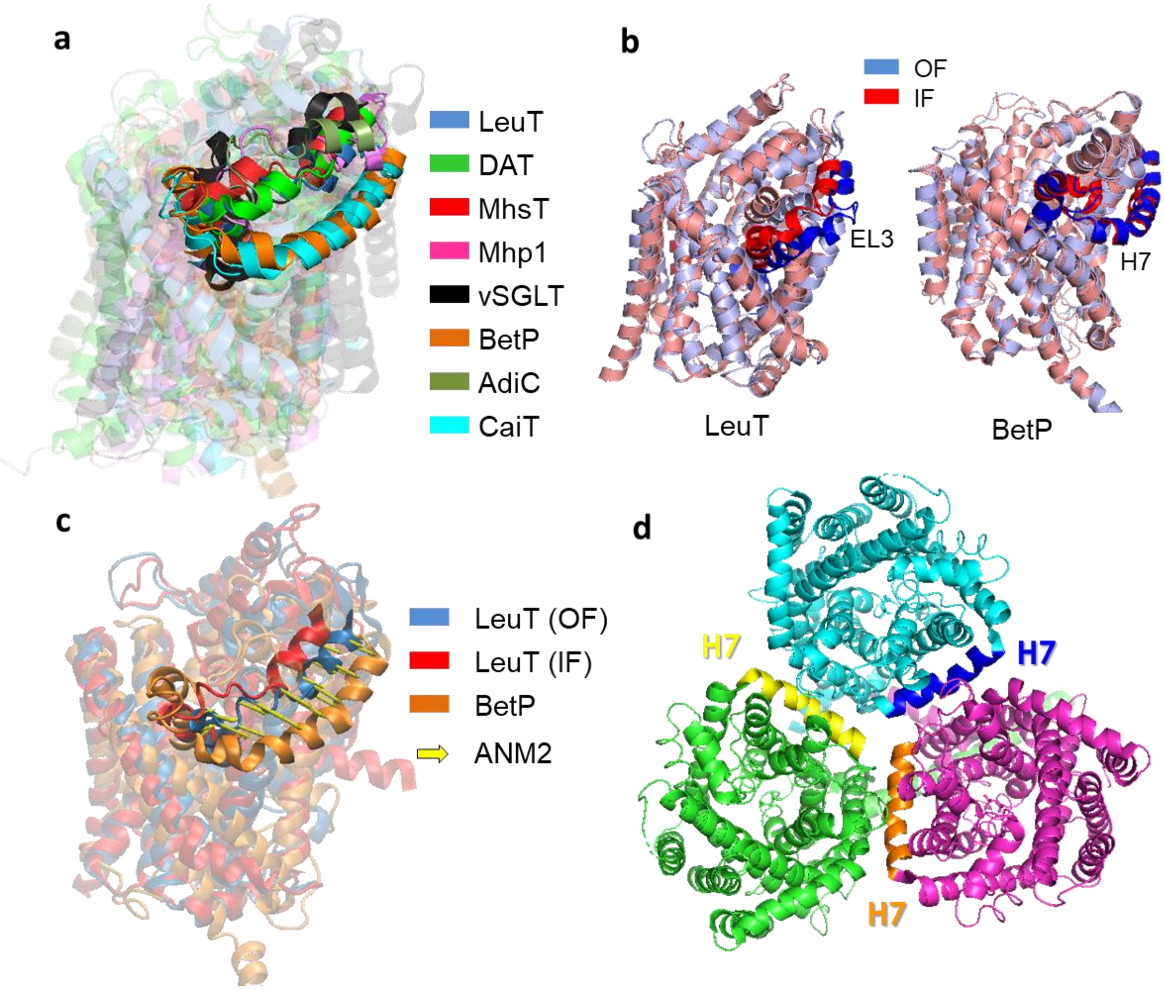

Supplementary Fig. S9. Structural alignment of LeuT family members reveals the member-specific characteristics of LeuT EL3 and BetP H7 and their role in alternating access and multimerization. (**a**) Superposition of representative structures from each family. The region of LeuT EL3 or equivalent BetP H7 is highlighted. BetP and CaiT (both trimeric) are distinguished from other members of the LeuT fold family. (**b**) Comparison of the OF and IF structures of LeuT and BetP, respectively. (**c**) Alignment of LeuT OF and IF structures and a BetP structure. The structural change in this region, predicted by ANM mode 2 calculated on a single LeuT OF structure (PDB ID: 2A65), consistent with earlier computations (Cheng and Bahar 2013; Ponzoni, et al. 2018), is indicated by the *yellow arrows*. (**d**) BetP trimer with each protomer coloured differently and the H7 helices highlighted by a different colour.

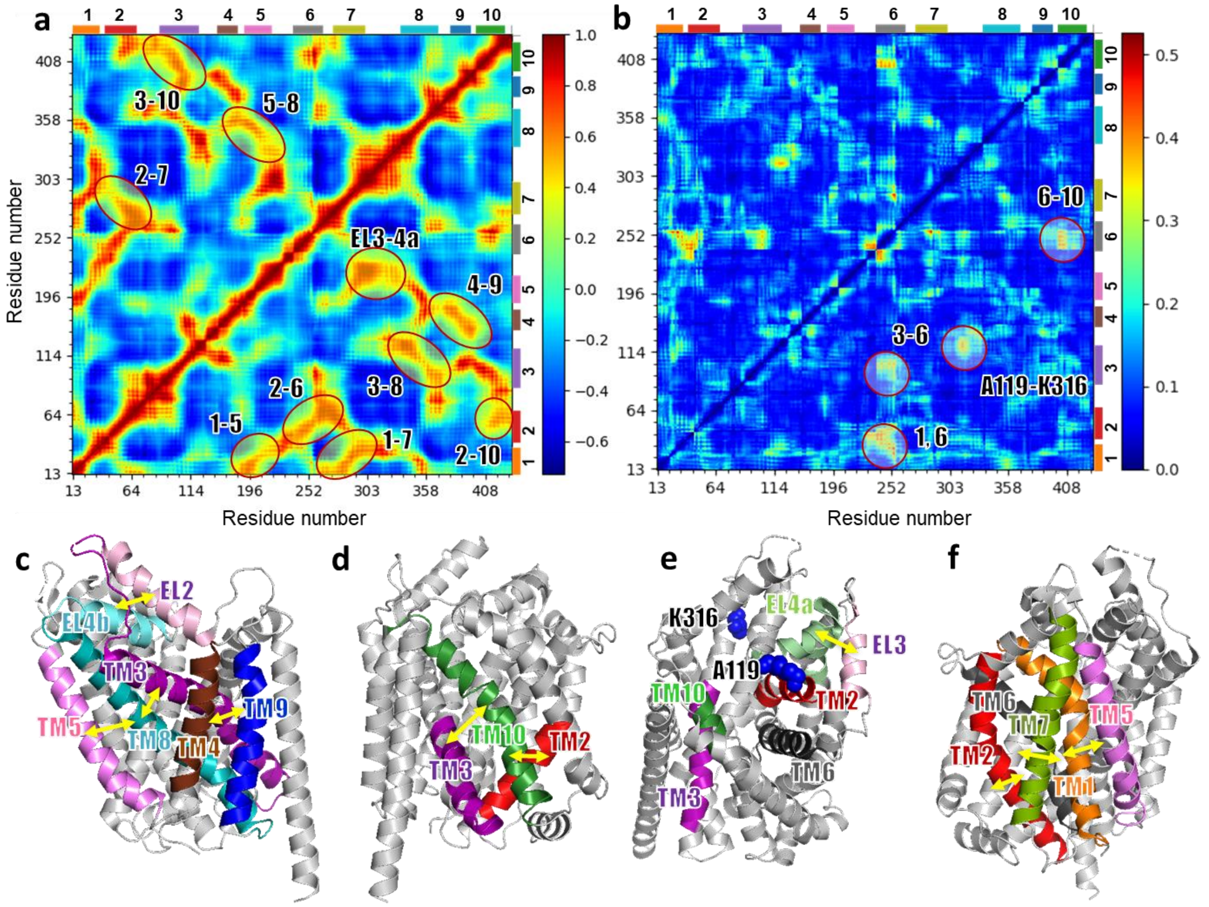

Supplementary Fig. S10. Conserved and differentiated couplings between the dynamics of LeuT fold TM domains and EC loops. (**a**) Generic covariance matrix characteristic of the LeuT fold. Cross-correlations averaged over LeuT fold family members are presented. High cross-correlations, indicative of conserved and strong couplings between pairs of regions, are highlighted by *ellipses*, and labels indicate the corresponding transmembrane helix indices. We note among them strong couplings between TM1, 7 and 5 (*red*), while TM1 and TM6 are anticorrelated (*blue*). (**b**) Standard deviations in the cross-correlations. The regions exhibiting the highest deviations are highlighted by *circles*. (**c-e**) TM helices exhibiting conserved couplings (panel **a**) in their dynamics are coloured and labelled, shown from different perspectives. The couplings between the pairs of helices are indicated by *yellow arrows*. Ribbon diagrams were constructed using the reference OF LeuT structure – the first structurally resolved member of the LeuT fold family (PDB ID: 2A65). (**f**) Relative positions of TM helices highlighted in panel **b**, which exhibit significant departures from the generic behaviour. We note in particular TM1-TM6, TM3-TM6 and TM6-TM10 pairs which exhibit significant departures from the average behaviour, predominantly due to the high mobility of TM6.

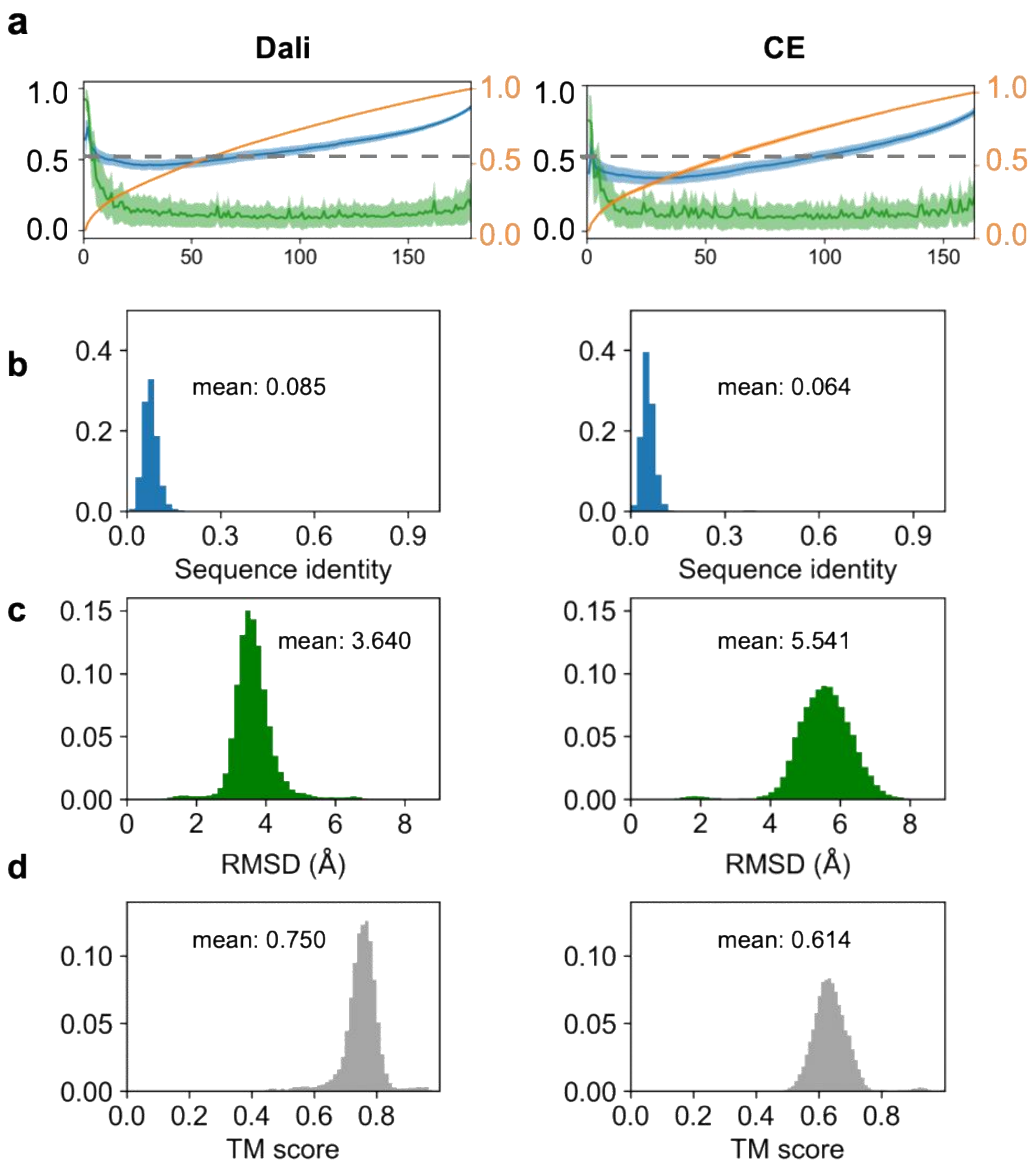

Supplementary Fig. S11. A better alignment of structure ensemble yields better mode overlaps, and distributions of sequence and structural metrics, but the overall trends are maintained. (**a**) Mode conservation probability (*green*), cumulative spectral overlaps (*blue*), and cumulative weights (*orange*) of individual modes for TIM barrel fold structure ensemble (see **supplementary fig. S1** and **supplementary table S3**) aligned using the Dali alignment method (*left*) and the CE alignment method (*right*). The mode conservation probability and cumulative

spectral overlaps for the Dali alignment method for TIM barrel fold structure ensemble is higher than that with CE alignment method, especially in the LTIF modes. Distributions of (**b**) sequence identities, (**c**) RMSDs and (**d**) TM scores of the TIM barrel fold structures for the Dali (*left*) and CE (*right*) alignment methods. All results were generated structure ensembles containing the 290 PDB chains in **supplementary table S3**.

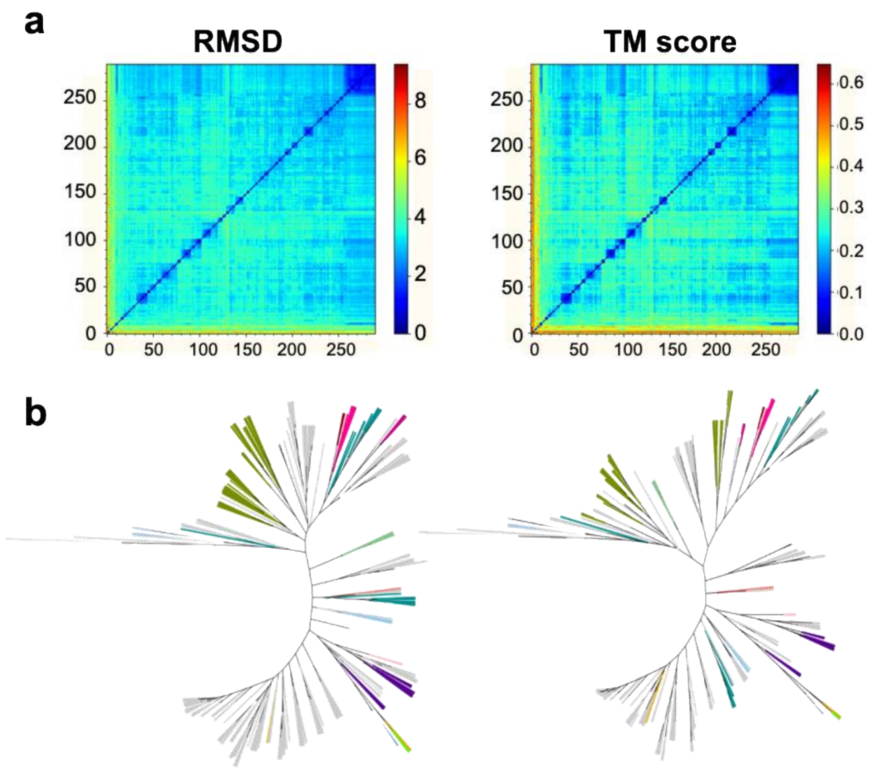

Supplementary Fig. S12. TM score produces similar results to RMSD for TIM barrel fold structures. (**a**) Distance matrices and corresponding (**b**) dendrograms for the Dali aligned TIM barrel fold structures based on RMSD (*left*) and 1 – TM score (*right*), respectively. In the trees, each clade represents a structure which is coloured based on CATH subfamilies as listed in **supplementary table S3**. The pairs of distance matrices and corresponding dendrograms based on two different metrics exhibit similar features.

Supplementary Tables

Supplementary tables S1-4 are presented in Excel files.

Supplementary Movies

Movie 1. PBP-1 signature ANM mode 1 observed from a view facing into the cleft. This mode features an inter-lobe twisting motion as indicated by the arrows.

Movie 2. PBP-1 signature ANM mode 2 observed from a view facing into the cleft. This mode features cleft opening and closing as indicated by the arrows.

Movie 3. LeuT fold signature ANM mode 1 observed from the extracellular side. The motion is obtained by taking the average of mode 1 calculated from each LeuT superfamily member.

Movie 4. LeuT fold signature ANM mode 2 from the extracellular side. The motion is obtained by taking the average of mode 2 calculated from each LeuT superfamily member.

Movie 5. LeuT fold signature ANM mode 3 from the extracellular side. The motion is obtained by taking the average of mode 3 calculated from each LeuT superfamily member.

Movie 6. ANM mode 2 of IF LeuT facilitates the transition from the closed (LeuT IF) to the extended (BetP) conformation. The highlighted region is EL3 for LeuT, DAT and MhsT, or equivalently H7 for BetP and CaiT. The ANM mode 2 is computed based on the LeuT OF structure.

Supplementary References

Ahmed A, Villinger S, Gohlke H. 2010. Large-scale comparison of protein essential dynamics from molecular dynamics simulations and coarse-grained normal mode analyses. Proteins 78:3341-3352.

Bahar I, Jernigan RL, Dill KA. 2017. Protein Actions: Principles and Modeling: Garland Science.

Banner DW, Bloomer AC, Petsko GA, Phillips DC, Pogson CI, Wilson IA, Corran PH, Furth AJ, Milman JD, Offord RE, et al. 1975. Structure of chicken muscle triose phosphate isomerase determined crystallographically at 2.5 angstrom resolution using amino acid sequence data. Nature 255:609-614.

Brüschweiler R. 1995. Collective protein dynamics and nuclear spin relaxation. J Chem Phys 102:3396-3403.

Cheng MH, Bahar I. 2013. Coupled global and local changes direct substrate translocation by neurotransmitter-sodium symporter ortholog LeuT. Biophys J 105:630-639.

Cock PJ, Antao T, Chang JT, Chapman BA, Cox CJ, Dalke A, Friedberg I, Hamelryck T, Kauff F, Wilczynski B, et al. 2009. Biopython: freely available Python tools for computational molecular biology and bioinformatics. Bioinformatics 25:1422-1423.

Dawson NL, Lewis TE, Das S, Lees JG, Lee D, Ashford P, Orengo CA, Sillitoe I. 2017. CATH: an expanded resource to predict protein function through structure and sequence. Nucleic Acids Res 45:D289-D295.

Dutta A, Krieger J, Garcia-Nafria J, Lee J, Greger IH, Bahar I. 2015. Cooperative dynamics in intact AMPA and NMDA glutamate receptors – similarities and subfamily-specific differences. Structure 23.

Fuglebakk E, Echave J, Reuter N. 2012. Measuring and comparing structural fluctuation patterns in large protein datasets. Bioinformatics 28:2431-2440.

Gur M, Zomot E, Bahar I. 2013. Global motions exhibited by proteins in micro- to milliseconds simulations concur with anisotropic network model predictions. J Chem Phys 139:121912.

Hess B. 2002. Convergence of sampling in protein simulations. Phys Rev E Stat Nonlin Soft Matter Phys 65:031910.

Hinsen K, Petrescu A-J, Dellerue S, Bellissent-Funel M-C, Kneller GR. 2000. Harmonicity in slow protein dynamics. Chem Phys 261:25-37.

Holm L, Laakso LM. 2016. Dali server update. Nucleic Acids Res 44:W351-W355.

Ilgü H, Jeckelmann J-M, Gapsys V, Ucurum Z, de Groot BL, Fotiadis D. 2016. Insights into the molecular basis for substrate binding and specificity of the wild-type L-arginine/agmatine antiporter AdiC. Proceedings of the National Academy of Sciences 113:10358-10363.

Jin R, Singh SK, Gu S, Furukawa H, Sobolevsky AI, Zhou J, Jin Y, Gouaux E. 2009. Crystal structure and association behaviour of the GluR2 amino-terminal domain. EMBO J 28:1812-1823.

Kalayil S, Schulze S, Kuhlbrandt W. 2013. Arginine oscillation explains Na+ independence in the substrate/product antiporter CaiT. Proc Natl Acad Sci U S A 110:17296-17301.

Kuhn HW. 1955. The Hungarian method for the assignment problem. Naval Research Logistics (NRL) 2:83-97.

Larkin MA, Blackshields G, Brown NP, Chenna R, McGettigan PA, McWilliam H, Valentin F, Wallace IM, Wilm A, Lopez R, et al. 2007. Clustal W and Clustal X version 2.0. Bioinformatics 23:2947-2948.

Leioatts N, Romo TD, Grossfield A. 2012. Elastic Network Models are Robust to Variations in Formalism. J Chem Theory Comput 8:2424-2434.

Letunic I, Bork P. 2016. Interactive tree of life (iTOL) v3: an online tool for the display and annotation of phylogenetic and other trees. Nucleic Acids Res 44:W242-W245.

Malinauskaite L, Quick M, Reinhard L, Lyons JA, Yano H, Javitch JA, Nissen P. 2014. A mechanism for intracellular release of Na+ by neurotransmitter/sodium symporters. Nat Struct Mol Biol 21:1006-1012.

Ming D, Wall ME. 2005. Allostery in a coarse-grained model of protein dynamics. Phys Rev Lett 95:198103.

Murzin AG, Brenner SE, Hubbard T, Chothia C. 1995. SCOP: a structural classification of proteins database for the investigation of sequences and structures. J Mol Biol 247:536-540.

Perez C, Faust B, Mehdipour AR, Francesconi KA, Forrest LR, Ziegler C. 2014. Substrate-bound outward-open state of the betaine transporter BetP provides insights into Na+ coupling. Nat Commun 5:4231.

Ponzoni L, Zhang S, Cheng MH, Bahar I. 2018. Shared dynamics of LeuT superfamily members and allosteric differentiation by structural irregularities and multimerization. Philos Trans R Soc Lond B Biol Sci 373.

Romo TD, Grossfield A. 2011. Validating and improving elastic network models with molecular dynamics simulations. Proteins 79:23-34.

Saitou N, Nei M. 1987. The neighbor-joining method: a new method for reconstructing phylogenetic trees. Mol Biol Evol 4:406-425.

Shindyalov IN, Bourne PE. 1998. Protein structure alignment by incremental combinatorial extension (CE) of the optimal path. Protein Eng 11:739-747.

Simmons KJ, Jackson SM, Brueckner F, Patching SG, Beckstein O, Ivanova E, Geng T, Weyand S, Drew D, Lanigan J, et al. 2014. Molecular mechanism of ligand recognition by membrane transport protein, Mhp1. EMBO J 33:1831-1844.

Sokal RR. 1958. A statistical method for evaluating systematic relationship. University of Kansas science bulletin 28:1409-1438.

Wang KH, Penmatsa A, Gouaux E. 2015. Neurotransmitter and psychostimulant recognition by the dopamine transporter. Nature 521:322-327.

Watanabe A, Choe S, Chaptal V, Rosenberg JM, Wright EM, Grabe M, Abramson J. 2010. The mechanism of sodium and substrate release from the binding pocket of vSGLT. Nature 468:988-991.

Yamashita A, Singh SK, Kawate T, Jin Y, Gouaux E. 2005. Crystal structure of a bacterial homologue of Na+/Cl--dependent neurotransmitter transporters. Nature 437:215-223.

Zheng W, Brooks BR. 2005. Probing the local dynamics of nucleotide-binding pocket coupled to the global dynamics: myosin versus kinesin. Biophys J 89:167-178.
